# Generating synthetic population for simulating the spatiotemporal dynamics of epidemics

**DOI:** 10.1371/journal.pcbi.1011810

**Published:** 2024-02-12

**Authors:** Kemin Zhu, Ling Yin, Kang Liu, Junli Liu, Yepeng Shi, Xuan Li, Hongyang Zou, Huibin Du

**Affiliations:** 1 Shenzhen Institute of Advanced Technology, Chinese Academy of Sciences, Shenzhen, China; 2 Hangzhou Institute of Technology, Xidian University, Hangzhou, China; 3 College of Management and Economics, Tianjin University, Tianjin, China; 4 National Industry-Education Platform of Energy Storage, Tianjin University, Tianjin, China; University of Washington, UNITED STATES

## Abstract

Agent-based models have gained traction in exploring the intricate processes governing the spread of infectious diseases, particularly due to their proficiency in capturing nonlinear interaction dynamics. The fidelity of agent-based models in replicating real-world epidemic scenarios hinges on the accurate portrayal of both population-wide and individual-level interactions. In situations where comprehensive population data are lacking, synthetic populations serve as a vital input to agent-based models, approximating real-world demographic structures. While some current population synthesizers consider the structural relationships among agents from the same household, there remains room for refinement in this domain, which could potentially introduce biases in subsequent disease transmission simulations. In response, this study unveils a novel methodology for generating synthetic populations tailored for infectious disease transmission simulations. By integrating insights from microsample-derived household structures, we employ a heuristic combinatorial optimizer to recalibrate these structures, subsequently yielding synthetic populations that faithfully represent agent structural relationships. Implementing this technique, we successfully generated a spatially-explicit synthetic population encompassing over 17 million agents for Shenzhen, China. The findings affirm the method’s efficacy in delineating the inherent statistical structural relationship patterns, aligning well with demographic benchmarks at both city and subzone tiers. Moreover, when assessed against a stochastic agent-based Susceptible-Exposed-Infectious-Recovered model, our results pinpointed that variations in population synthesizers can notably alter epidemic projections, influencing both the peak incidence rate and its onset.

## Author summary

Exploring the intricacies of infectious disease transmission becomes profoundly insightful with tools that can model complex, nonlinear interactions. Among these, agent-based models stand out, primarily due to their ability to mirror not just expansive populations but also detailed individual-level interactions. While synthetic populations act as a vital surrogate for the real-world demographic when comprehensive datasets are elusive, their creation isn’t without challenges. Present synthesizers, though acknowledging intra-household relationships, still have nuances left unexplored. Addressing this, our research presents a pioneering method tailored for crafting synthetic populations, especially for disease spread simulations. This methodology, grounded in data from genuine household structures, utilizes an optimization strategy to calibrate these relationships effectively. Applying this, we meticulously constructed a synthetic population for Shenzhen, China, encompassing over 17 million agents. Our results underscored the technique’s prowess in accurately emulating population structures, adhering commendably to demographic metrics on both city and subzone scales. Crucially, our findings also illuminated that the specific choice of synthesizer can have a profound bearing on epidemic simulations, affecting crucial attributes like the peak and timing of disease incidence.

## 1. Introduction

Agent-based microsimulation models are gaining in significance in the domain of epidemic modeling [1–4]. Compared to traditional aggregated simulation models (i.e., meta-population models), agent-based models simulate the attributes and behaviors of each individual over time, offering comprehensive and precise insights into the transmission of epidemics [5].

Nearly all agent-based models employ a synthetic population of households and individuals with relevant attributes. Census data from a city is an optimal data source for this purpose, as it contains fully disaggregated information pertaining to the entire population. However, collecting such data could be subject to specific restrictions, such as confidentiality and, importantly, costs [6]. Therefore, creating synthetic population data is deemed a cost-effective solution to furnish agent-based simulation models with synthetic populations that are reasonably accurate.

The objective of the population synthesis algorithm is to utilize a small sample of the population, such as data obtained from a household travel survey, to establish a model from which the complete population can be generated based on certain assumptions. Significant research has already been performed in the fields of transportation simulation and urban planning, and there are many well-developed tools for population synthesis modeling, which can provide a list of agents with various sociodemographic attributes (age, gender, occupation, income, or whether the agent has a car/driving license, etc.) for urban planning/ transportation microsimulations [7–10].

Unlike the models described above, the agent-based model for epidemic simulation focuses on the different agent attributes of interest. For example, agent probabilities of morbidity, severe illness, and death after infection are usually age-dependent when modeling respiratory diseases. Thus, age-related attributes are extremely important for expressing agent heterogeneity in these models. Although some studies [11] indicate that income also affects the dynamics of disease transmission, in this study, our focus in the modeling is placed on the age structure factors within the synthetic population. Consequently, attributes such as income have not been included as individual attributes in our model.

Several studies have revealed that the household structure has a profound impact on the transmission of infectious diseases [12–14]. Although previous research has attempted to associate both household- and individual-level attributes in a unified manner, they most simply fit the marginal distribution of household-level attributes (e.g., household size), and thus fail to capture and reproduce the interdependencies among agents within the same household [15]. Unfortunately, this may mean that some significant patterns are overlooked. For example, a household comprises three generations: children, parents, and grandparents. Children are more likely to be infected with pathogens, particularly those causing fecal-orally transmitted [16] and respiratory diseases [17], due to their social activities and frequent contact with children in the same age group and can transmit these infections to their grandparents [18]. This particular transmission pattern can lead to outbreaks in the elderly population during an epidemic. However, models cannot capture and reproduce this phenomenon unless the interdependencies among household members are preserved in the synthetic population. This introduces potential bias into the epidemic simulation process. and affects the output of the model, which in turn misleads decision makers. Therefore, population synthesis for epidemic simulation sets higher requirements for the representativeness of the household structure.

Another important distinction between the general population synthetic model and the design of an epidemic simulation is the explicit representation of the locations of individuals. While spatial models of disease transmission are important in understanding the spread of epidemics, it is worth noting that in most studies, spatial population data is primarily utilized for mapping epidemics rather than projecting the risk of infection at an individual level [19]. Traditionally, spatial representation of synthesized agents in daily activity chains either attach to individuals based on sociodemographic attributes or are constructed by applying activity-based models [20–22]. In this study, we focus on the demographic attributes of individuals and households, rather than their mobility behavior. This focus has guided our method of representing individuals’ geographic locations at the zonal level of their residential addresses. By this approach, we effectively generate a synthetic population dataset with spatial regional labels, suitable for epidemic simulation analysis, without involving detailed activity logs or travel trajectories of each individual for downstream tasks.

The present study was performed with the aim of providing a population synthesis framework that can capture and reproduce the structure and spatial distribution of populations at both the household and individual levels. To more comprehensively capture the interdependencies between individual and household characteristics, the proposed framework integrates two stages: (1) typical household structure selection, which is applied to capture the most common household types and their frequency from a small fraction of disaggregated surveys; and (2)combinatorial optimization, which generates a whole synthetic population composed of combinations of household structures that consist of the marginal distributions for attributes of full census data. These two steps ensure that our synthetic population preserves the underlying interdependence among individuals within the same household based on their structural relationships. Further, an agent-based Susceptible-Exposed-Infectious-Recovered (SEIR) model was constructed to allow qualitative assessment of the impact of different population synthesis methods on epidemic modeling.

## 2. Literature review

In this section, we first reviewed the current state of population synthesis methods. Then, we delved into the use of synthetic populations in infectious disease models. Finally, we examined the limitations of previous studies and proposed some of the contributions this paper can make to the literature.

### 2.1 Population synthesis methods

Population synthesis modeling has a rich and extensive literature, with the Iterative Proportional Fitting (IPF) algorithm standing out as the most widely adopted approach. Originally developed as a method for adjusting contingency tables [23], it is a log-linear model that preserves only the main effects, and has been applied in various fields such as urban studies and transportation research. Despite its popularity, there are limitations to the IPF model that researchers are continuously working to address. For instance, the "zero-cell problem" occurs when there are attributes with limited or no observations, which can be addressed using sparse matrix manipulation techniques as proposed by [24]. Additionally, the IPF method can become computationally burdensome when dealing with a large number of attributes, particularly those with multiple categories, which can limit scalability [25,26].

In terms of population synthesis for microscope epidemic simulation, the most notable limitation of the IPF is that it matches distributions only at a single demographic level; thus, it is unable to associate household- and individual-level attributes in a unified manner [27]. The Iterative Proportional Updating algorithm proposed by [28] aimed to match household and person attributes as closely as possible in a universal generator. This algorithm allows for simultaneous control of both levels to better control the fitting at both the household and individual levels. In addition, hierarchical and multistage IPF procedures have been proposed to preserve these relationships [29,30]. PopGen, an open-source synthetic population generator [31], was implemented using this algorithm.

Population synthesis can be classified into two main approaches based on whether the goal is to create the attributes of entities or to replicate known real entities. These two approaches are commonly referred to as Synthetic Reconstruction (SR) and Combinatorial Optimization (CO), respectively [31]. CO is frequently used for population synthesis and it attempts to generate an optimized solution by randomly selecting samples from the microdata while minimizing the differences in marginals using algorithms such as Simulated Annealing [32,33]. CO-based methods is characterized by the replication of existing agents in microsamples [34]. Other variations of CO, such as fitness-based methods [35], follow the process of microsample replication. However, as previously mentioned, an over-reliance on replication can result in several conceptual and empirical challenges.

In contrast, SR is a population synthesis approach that leverages both detailed and summarized data to reconstruct individual entities, relying on the estimation of the most accurate underlying attribute distribution. Initially, this technique relies on granular data, often a sample, which is assumed to be a representative subset of the overall population, commonly referred to as seed data. Subsequently, the synthetic population is created by allocating individuals with specific socio-demographic characteristics to designated areas. This allocation employs a weighting mechanism to align the marginal distribution with aggregated data, typically derived from comprehensive sources like census data. One established approach for achieving this alignment is through deterministic re-weighting algorithms [36,37]. These algorithms assign weights to individual records within the granular data, treating them as probability distributions derived from the available aggregated data. The process treats each attribute of the population units independently, performing sampling from marginal distributions to select units matching the area-specific population totals.

The literature review in this study primarily focuses on conventional population synthesis methods. However, it is important to acknowledge that more advanced methods have emerged in recent years, which warrant attention. For instance, probabilistic graph models, including Hidden Markov Models (HMMs) and Bayesian Networks, have been explored for population synthesis tasks [34,38,39]. These models offer a probabilistic framework for populations synthesis, allowing for the incorporation of uncertainty and capturing complex dependencies among attributes. Moreover, deep generative models, such as Generative Adversarial Networks (GANs) and Graph Autoencoders (GAEs), have gained significant traction in various data synthesis applications, including population synthesis [40]. These models have the capacity to generate diverse and realistic synthetic populations, potentially offering advantages over traditional methods. Additionally, the concept of ‘spatially explicit’ population synthesis has received attention, enabling the creation of synthetic populations that capture geographic distribution patterns [41]. However, these deep learning models often require extensive hyperparameter tuning and training, adding complexity and reducing practicality in implementation. Thus, we chose CO methods due to their typically lightweight algorithmic structures, making them easier to implement and highly efficient when handling large population data. This lightweight structure and ease of implementation give our approach practical relevance in the field of infectious disease modeling and facilitate its adoption and application by other modeller.

It is worth noting that while previous methods have been instrumental, the challenge of accurately reproducing structural relationships among household members persists. These structural relationships are closely linked to individual interactions within households and can significantly impact the modeling of infectious diseases. Introducing this type of household structural information into population synthesis models has the potential to enhance our ability to capture these intricate network structures.

### 2.2 Epidemic simulation with synthetic population

Epidemic simulation comprises a related and often overlapping stream of research that requires a highly realistic synthetic population. To simulate the spread of infections in populations with different geographic and demographic characteristics, the modeler typically uses census data, either from the population they are planning to recreate or from a similar population [3,42]. Synthetic populations have been widely recognized as a valuable tool for epidemic modeling in numerous studies [26,43–46]. This is particularly important when population heterogeneities play a critical role in disease transmission [47], or when disease incidence or infection risk varies significantly across subgroups, such as age groups [48–50]. Recent findings regarding the highly variable prevalence of COVID-19 across age groups [51,52] have further underscored the pivotal role of demographics in shaping transmission dynamics.

Compared with research on urban planning or transportation models, population synthesis for epidemic modeling focuses on different scientific issues and attributes of interest. However, to the best of our knowledge, there are only a few population synthesis methods dedicated to the design of agent-based epidemic modeling, including the population synthesis module within epidemic modeling toolkits [53]. Some existing epidemic agent-based models use real-world data from surveys to model population and inter-agent contacts within small regions, such as blocks [1] or campuses [54]. However, this approach involves privacy concerns and also limits the scope of application of the model to a small study area. When it comes to modeling the development of epidemics at the city level or even larger research scales, this kind of method is not applicable in terms of either cost or data accessibility.

When modeling epidemic transmission among large populations lacking real-world data, various techniques have been applied to existing agent-based models to cope with the population synthesis problem. Given its simplicity, IPF has become the primary choice in population synthesis for various types of realistic modeling problems [55,56], including epidemic modeling [57,58]. Neglecting household structure information may introduce potential bias into the constructed synthetic population and consequently influence the simulation of the disease transmission process.

As an alternative approach to generating synthetic populations for microscale epidemic simulations, some agent-based models make use of open-source modules or software originally designed for and extensively applied in transportation, ecology, and urban planning research. Examples of such tools include *TransSim* and *MATSim* [59,60], as well as other tools like SPEW (implemented in R language) [61] and Gen* (implemented in Java language) [62], which are also utilized for this purpose. These open-source platforms provide practical tools for creating a representative synthetic population for urban transportation planning and can be readily adopted for epidemic modeling. Most of these platforms have the capability to link household-level and individual-level attributes in a cohesive manner. Some activity-based travel demand models such as *ActivitySim* can simulate the travel choices/trips of individuals based on classic transportation simulators such as the four-step travel model. Although they have been widely applied in the field of microscopic epidemic modeling, general-purpose population synthesizers are complicated and computationally intensive. The task of characterizing joint associations among a large set of attributes becomes challenging due to the curse of dimensionality, particularly when these attributes are organized hierarchically at both the household and individual levels [15]. Fitting socioeconomic attributes including income, building type, and number of vehicles at both household and individual levels may impose a heavy and unnecessary computational burden, as these attributes are not of interest in most epidemic research. Owing to computational insensitivity, most agent-based simulators for epidemic modeling tend to generate a sampled synthetic population instead of a population with the actual size of the study area, potentially weakening the representation of the synthetic population.

In conclusion, current population synthesizers focus on fitting multidimensional socioeconomic attributes and, therefore, fail to effectively capture the relationships among household members, leading to potential biases in agent-based simulations of infectious disease transmission based on synthetic agents. As such, there is a strong need to construct an alternative population synthesis framework that can accurately reflect the distribution of household structures in the population and thus characterize the underlying distribution of interdependency.

## 3. Materials and methods

### 3.1 Framework

**Fig 1:**
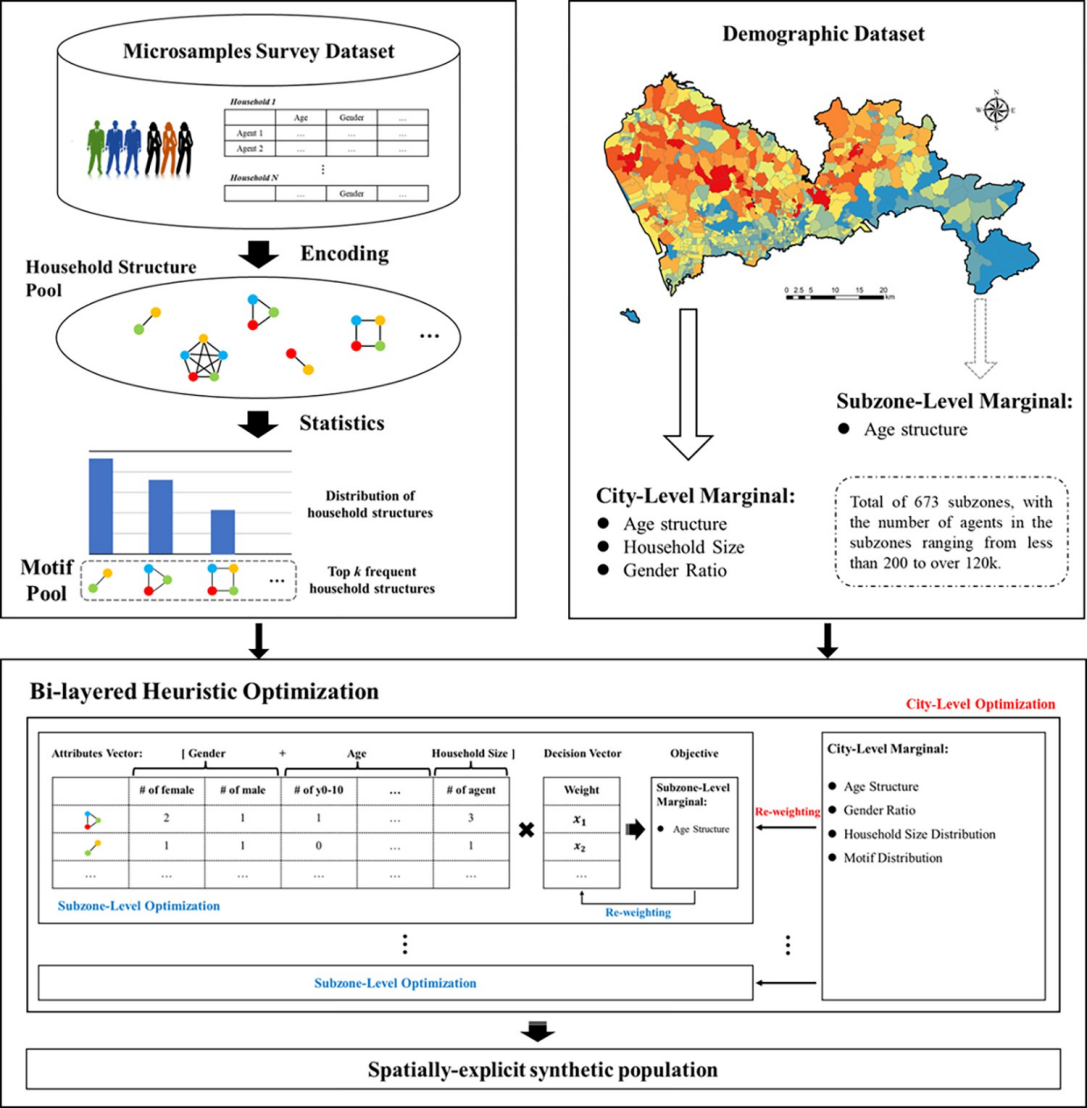
The framework of population synthesis. **The basemap shapefile can be accessed at** *https*:*//www*.*kaggle*.*com/datasets/keminzhu/basemap-shenzhen-subzones*.

In this section, we present the framework for population synthesis (Fig 1), with a focus on designing an epidemic simulation using a household motif optimization model. This procedure consists of two phases: motif selection and optimization. In the selection phase, we prioritized understanding the ’co-inhabitant’ that could lead to disease transmission among household members. We generated a finite pool of age-specific structures, known as household motifs, to represent the most common household types. These motifs were generated using a data-driven approach, analyzing the statistical patterns of encoded household structures from microsample survey data. Subsequently, in the optimization phase, we employed a heuristic algorithm to adjust the weights assigned to these household motifs. This process aimed to generate a synthetic population that matched the marginal attribute distributions in census data at both the city and subzone levels. Using this synthetic population, which represented as a list of agents, we established a stochastic agent-based epidemic model to assess how generated synthetic populations impacts disease transmission simulations.

### 3.2 Preprocessing and encoding of the household structure

One essential input for this problem is the public use of micro samples (PUMS); here, we use the household and travel survey data of Shenzhen as an alternative because the PUMS is not available. Table 1 presents an example dataset.

**Table 1:** Sample of household survey data.

HID	Np	Dwell	BuildingID	Car	PID	Age	Gender	Occupation	PhoneOwner
1	2	FH	0400****1700000	Yes	1	42	Male	22	Yes
	2	FH	0400****1700000	Yes	2	36	Female	5	Yes
2	1	CD	0700****4200000	No	3	33	Male	14	Yes
3	1	CD	0701****2300000	No	4	29	Male	21	Yes
…	…	…	…	…	…	…	…	…	…
*i*	$x_{i}^{1}$	$x_{i}^{2}$	$x_{i}^{3}$	$x_{i}^{4}$	$x_{i}^{5}$	$x_{i}^{6}$	$x_{i}^{7}$	$x_{i}^{8}$	$x_{i}^{9}$
…	…	…	…	…	…	…	…	…	…

*HID* and *PID* represent household and person ID, respectively. *Np* denotes the total number of individuals in the household. *Dwell* is a dwelling type (*FH*-family household/*CD*-collective dwell). In the following steps, only HID, PID, age, and sex were reserved.

Table 1 shows the raw survey data sample, in which we treated both household- and individual-level attributes as nominal categorical variables during the preprocessing stage. Each agent was divided into several age groups. To ensure simplicity, we arbitrarily classify a person’s age into ten-year groups to illustrate this subprocess. In practice, researchers can reset the age groups to satisfy the requirements of their simulation model. Socioeconomic attributes such as family income or whether an individual has a driving license were omitted from this study because those attributes are generally not considered critical variables in epidemic simulation. This helps to avoid unnecessary computation of irrelevant attributes and prevents the curse of dimensionality that transportation-oriented population synthesis models often suffer from.

Herein, $p_{i}^{k}$ denotes the *kth* member within the *ith* household *H*_*i*_ in the survey data, where *i* = 1,…,*m* and *k* = 1,…,*n*. We categorized each individual in the list into different types according to sex and age. For example, $p_{i}^{k}\in \{F,2\}$ for a female ages between 20-29(F/M stands for female/male). The household structure was subsequently encoded by counting the total number of each type of individual $p_{i}^{k}$ within the household. For example, given a family that consists of a 20–29 age couple (in the second age group) and two daughters under 10 (in the first age group) in the survey data, in the process of encoding, this family is marked as a unique household structure coded as”*(M*,*2)+(F*,*2)+(F*,*1)+(F*,*1)”*.

### 3.3 Household motif selection

There are 18 distinct individual-type $p_{i}^{k}$ under the current age group settings, which could result in over 47 millions possible household structures and 4729 in the dataset, even when we assume that the household size is no larger than six. This number increases exponentially if a finer age group is adopted, making subsequent optimization impossible within reasonable computational times. However, the majority of these household structures are rarely observed in real-world populations. For example, the household structure coded as”*(F*,*8)+(F*,*8)+(F*,*8)+(F*,*8)+(F*,*8)+(F*,*8)”* (a family consisting of six elderly females) would be infrequent in most cities and, therefore, can be ignored without losing substantial representativeness of the population.

Based on this intuition, we selected the top *S* most frequent household structures in the survey data as the approximate representation of the entire population. We call these household structures motifs in analogy to motifs in complex networks [63] and human mobility modeling [64], which refers to a distinctive, usually recurrent structural element.

Specifically, we first traverse the survey data and record the household structures. These household structures were then reranked based on how often they appeared. Given a representative threshold *α*, one can obtain a minimum *S* to ensure that

$$\sum P({HS}_{i})\geq \alpha ,i=1,\ldots ,S,$$

where *HS*_*i*_ is the *ith* household structure after re-ranking, *P*(*HS*) is the frequency of *HS* in the survey data. These *HS*_*i*_ motifs were selected for subsequent optimization. During the traversal process, *P*(*HS*) was recorded as the initial value of the following optimization procedure (Table 2).

**Table 2:** An example of a household motif weight matrix.

*Motif*	*N*_*p*_	*N*_*f*_	*N*_*m*_	*N*_*a*0_	*N*_*a*1_	*N*_*a*2_	*N*_*a*3_	…	*X*_*init*_
(M,3)+(F,2)	2	1	1	0	0	1	2	…	0.071
(M,2)+(F,2)	2	1	1	0	0	2	0	…	0.063
(M,3)+(F,3)	2	1	1	0	0	0	3	…	0.060
(M,2)+(F,3)	2	1	1	0	0	1	2	…	0.048
(M,4)+(F,3)	2	1	1	0	0	0	2	…	0.045
(M,3)	1	0	1	0	0	0	1	…	0.034
(M,0)+ (M,3)+(F,3)	3	1	2	1	0	0	2	…	0.008
**…**	…	…	…	…	…	…		…	…

*N*_*p*_ represents the number of individuals in a household, *N*_*f*_ and *N*_*m*_ represent the number of male and female individuals respectively, *N*_*ak*_ represents the number of individuals in the *k*-th age group, *X*_*init*_ represents the frequency of the household structure *P*(*HS*) in the survey data and serves as the initial value for optimization.

Another major limitation of this motif-selection method is that it captures only the existing household structure in the household survey data, which means that If a particular combination of attributes for a household, such as a specific composition of agents, is not observed in the survey data, it may not be generated in the synthetic population because the corresponding cell in the initial distribution is zero [15]. This zero-cell problems may arise during the integration of population census demographics with household survey data. These issues are primarily associated with conventional IPF/CO methods and can occur when the nominal categories are overly fine-grained, and the sample survey data lack sufficient coverage for all possible attribute combinations. To address this problem, we replace incorrect zero-cell values with small positive values (e.g., 10^−5^). This approach has been commonly used in previous combinatorial optimization-based approaches [8].

### 3.4 Household motif combinatorial optimization

In order to generate the synthetic population for the epidemic simulation, we adjust and reallocate the weights among the household motifs selected in Section 3.3. The most commonly used method for this process is the Iterative Proportional Fitting (IPF) procedure, which involves estimating joint distributions that match the given marginal frequency distributions. However, this approach can lead to an inconsistency between weights for matching household- and individual-level distributions, even with more sophisticated algorithms [28]. Additionally, accurately characterizing household structures is a difficult task. Therefore, we propose an approach called the Motif Heuristic Optimization (MHO) to generate synthetic populations by incorporating the distribution of household motifs.

The algorithm begins by creating an attribute matrix *D* that contains all motifs and data describing the composition of the household (as shown in Table 2 above), where *N*_*p*_ is the household size of this motif, *N*_*f*_, *N*_*m*_, *N*_*a*k_ stand for the number of agents of different genders and age groups in this type of household. *X* denotes the weights of household motifs that are initialized by the frequency of observations in survey data $\tilde{X}$, and the target vector $\tilde{Y}=\{Y_{N},Y_{f},Y_{m},Y_{a0},Y_{a1},\ldots ,Y_{a8}\}$ denotes the values of the total size of the whole population and the number of agents of different genders and age groups in the census data that the motifs are to be re-weighted to match. This problem can be recast as an optimization problem, where the weights of household motifs *X* are decision variables or vectors. An objective function can be formulated to minimize the discrepancy between simulation *Y* = *D*∙*X* and observation $\tilde{Y}$ using mean square error. The mathematical formula is as follows:

$$Minimize:\;F(X)=\varphi (Y,\tilde{Y})+\tau (X,\tilde{X})$$

$$\mathrm{where}\;\varphi (Y,\tilde{Y})=\sum_{i=1}^{n}{\left(y_{i}-\tilde{y_{i}}\right)}^{2}/n$$

$$\tau (X,\tilde{X})=\sum_{i=1}^{m}{\left(\log x_{i}-\log\tilde{x_{i}}\right)}^{2}/m$$

Subjectto:0≤X≤ub

where the $\tau (X,\tilde{X})$ is the penalty term used to ensure that the optimized weights *X* are consistent with the weights of the household motifs observed in the survey data. We normalized this term using a logarithmic function to account for the significant power-law characteristics in the distribution of household motifs observed in the survey. *ub* is the upper bound vector of *X* which determines the search field of the optimization; in this case, *ub* is set as a full vector. An additional non-negative lower bound constraint is imposed on each to prevent negative weights, as negative persons or households obviously cannot exist.

After formulating the objective function and bound constraints, the objective of the optimization stage is to find the household structure that provides the best fit. Ideally, a straightforward method would involve enumerating all possible combinations of motifs and evaluating their scores. However, in practice, this is not feasible because the number of candidates increases exponentially with the number of selected motifs [65]. Therefore, to address this bound-constrained nonlinear minimization problem with acceptable computational efficiency, a trust region-reflective optimizer [66] was introduced to reweigh the motifs. As illustrated in Algorithm 1, This process can be viewed as an iterative search procedure, where the algorithm moves from one solution to a neighboring one until a stopping criterion is satisfied (Algorithm 1).

**Algorithm 1. Motif heuristic optimization procedure**

**ALGORITHM** Heuristic Optimization for Motif Re-weighting

INPUT: Attribute matrix ***D*** of motif pool; Motif distribution ***X***_***init***_; Maximum number of iterations to perform ***k***_***max***_, Absolute error ***ftol*** in between iterations that is acceptable for convergence; Known marginal vector $\tilde{Y}$

OUTPUT: Optimized motif weights vector ***X***

1: initialize $X\leftarrow \tilde{X},k\leftarrow 0$

2: **while** *k*<*k*_*max*_, **do**

3:  *X*_*k*_←*NEIGHBOR*(*X*)

4:  Δ*loss* = *loss*(*X*)−*loss*(*X*_*k*_), where $loss(X)=\sum \sqrt{D∙X-\tilde{Y}}+\delta (X-X_{init})$

5:  **if** Δ*f*<0, **then**

6:   update *X*←*X*_*k*_

7:  **end if**

8:  **if** |Δ*loss*|<***ftol***, **then**

9:   **break loop**

10:  **end if**

11: **end while**

Synthetic households can be generated and formed into a list of synthetic agents Based on the household motifs selected from the microsample survey data and their weights *X* obtained by heuristic optimization by repeating household motifs *X* times. However, it is worth noting that *X* obtained from the optimization process is a decimal number, which will be rounded off when generating the household list. This may introduce numerical bias and cause some infrequent household structures with low weights to be overlooked in the generated synthesis population. To address this problem, we generated synthetic populations by sampling with weights *X* as choice probabilities, instead of repeating household motifs. The necessity of this operation depends on the requirements of the various households in the downstream agent-based model.

## Results

This section demonstrates the performance of the population synthesis method proposed in the present study. In this example, a synthetic population of 17.37 million people among 673 communities was generated for Shenzhen, China, using a subsampled household survey and demographic data, and the effects of fitting the marginal distribution of the synthetic population and the joint distribution of gender-age combination at both city and subzone levels were tested. We subsequently compared the proposed model with two widely used population synthetic methods (Direct Inflating and Iterative Proportional Fitting) in terms of their ability to capture cross-age interdependency. The implications of this discrepancy in different synthetic populations for the simulation of infectious disease transmission are further discussed by conducting a simple *Susceptible-Infectious-Removed* microsimulation.

### 3.5 Data sources

#### (a) Household interviews

In 2016, the Shenzhen Land Transport Authority conducted a transportation survey that serves as a crucial data source for urban and transportation modeling/planning in China. Transportation surveys are typically used by urban/transportation planning agencies and research institutes to collect comprehensive demographic and socioeconomic information at both household and individual levels, along with a record of the trips/activities of each individual on a specific weekday. The entire survey dataset used here discovered 111,604 individuals from 46,001 households (approximately 1% of the total population).

Raw survey data were modified to fit the requirements of the model before application. For simplicity, the attributes of interest, including individual/household identifier, age, and gender, were reserved, whereas socioeconomic columns such as income, driving license, pass type, and social security status were discarded. Table 2 summarizes the household- and individual-level attributes from the modified survey. Additionally, the raw data contained collective dwellings such as staff quarters and school dormitory rooms. These non-family households were filtered based on their labels. The modified dataset comprised 29,698 households and 65,577 individuals. The average household size in the survey was 2.2, with the largest being 10. Regarding the age attribute, the census data were presented in numerical form; however, owing to missing data and the possibility of zero marginal occurrence, age was collated into nine groups, and the survey data were grouped accordingly.

It’s important to note that our data did not provide clear statistics on this type of co-habitation. Many groups within the data actually consisted of over a dozen individuals in non-traditional living arrangements, such as roommates flat-sharing or company dormitories, rather than strictly falling under the category of traditional family households. Introducing such data into our analysis could potentially introduce bias in subsequent epidemic simulations.

#### (b) Demographic data

The Demographic dataset was obtained from the seventh population census conducted in China since 2020, provided by the Shenzhen Municipal Bureau of Statistics. The dataset has a spatial resolution at the community level and contains the total population of each age group in all communities in Shenzhen, saved in tabular form with column names such as community name, population aged 0–10, population aged 11–20, population aged 21–30, etc.

The raw data contains 846 units by administrative division, which were then re-divided into 673 subzones ranging in size from 0.2–30 km^2^ by geographical boundaries. Data from uninhabited mountainous areas were discarded. The smallest and largest communities were *Nanao* and *TongSheng*, with fewer than 300 residents and over 120,000 residents, respectively.

### 3.6 Motifs selection and optimization

#### 3.6.1 Household structure analysis

**Fig 2:**
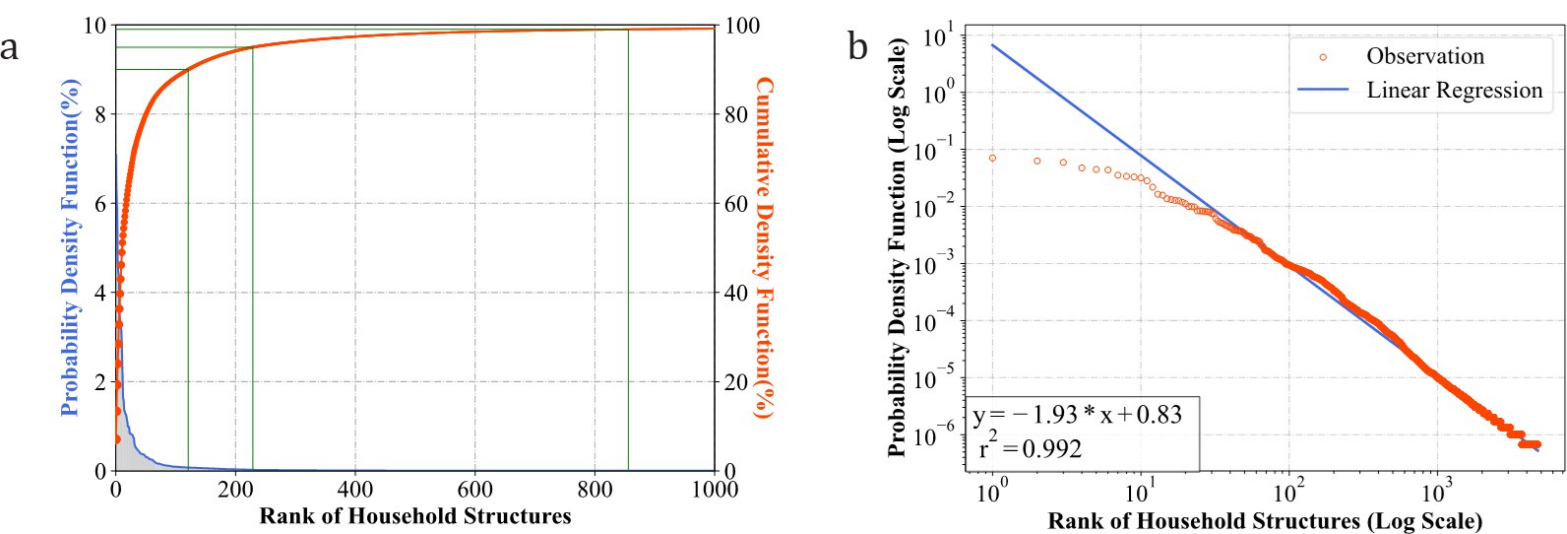
Distribution of household structure in survey data. (a) Probability/Cumulative Density Function. (b) Frequency-rank and Liner Regression.

The purpose of model selection was to obtain the most representative *S* household structures and their distributions from the microsample data. We, therefore, conducted statistical analyses on the distribution of coded household structures in the travel survey. Fig 2 illustrates the probability density and cumulative distribution functions of the 1000 most frequent household structures in the dataset.

The probability/cumulative density function curves illustrated in Fig 2(A) show that the top-ranked household structures are orders of magnitude different from other structures. For example, the most frequent household “*(M*,*3)+(F*,*2)*” accounted for 7% of all households in the survey, and only 124 structures were needed to cover over 90% of the households in the survey data, while 216 and 828 are needed to increase the coverage to 95% and 99%, respectively. A detailed list of the household structures and distributions is provided in the S1 Table.

To provide a more quantitative analysis, we projected the household distribution onto a double-log scale and applied linear fitting. As shown in Fig 2(B), the frequency of household structures in the survey exhibited a significantly truncated power-law distribution with *r*^2^>0.99. To further test the generalizability of this finding, we collected household-explicated survey data from provinces in China and other countries. The statistical and fitting results showed similar power-law distributions with different exponent metrics (*S2 Text*), indicating that this pattern is generalizable across geographic regions and populations.

#### 3.6.2 Optimization of motif weights

In this phase, the selected motifs were reweighted to satisfy the household- and person-level margin distributions, following the heuristic optimization procedure described in Section 3.5. The model starts iterating with the frequency of the distribution of different household motifs in the microsample data as an initial guess, and then continuously readjusts the weights of the motifs to achieve the optimizer objective. The objective function consisted of the residuals of the generated population at the margins of age, gender, and household size. The lower and upper bounds of the independent variables were set to zero and infinity, respectively, as the motif weight should not be negative. A linear loss function was applied to reduce the influence of outliers on the solution. The TRF algorithm was selected as the optimizer to perform minimization because it is particularly suitable for large sparse problems with bounds. The *Python* package *Scipy*. *optimize* was used to implement this process.

**Fig 3:**
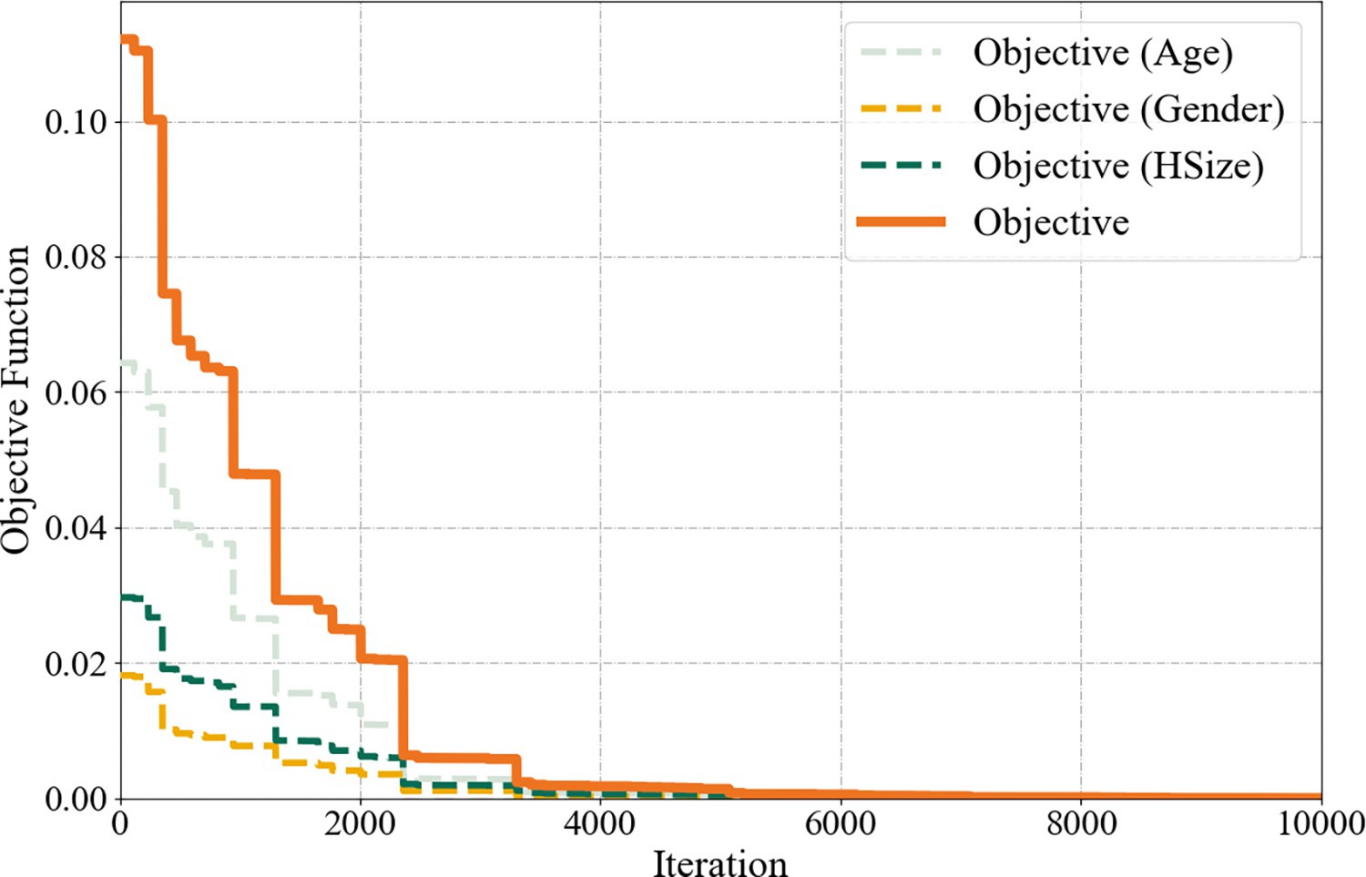
Changes in the objective function value with the iteration number.

Fig 3 shows how the objective function and components of the residuals for age, gender, and household size vary with an increasing number of iterations. During the initial stages of optimization, the objective function value was mainly contributed by the residuals of the distribution of age groups. Under the above experimental setup, the *Python* implementation takes approximately 0.2s per iteration and over 8000 iterations to finally converge (the relative error *ftol* in the objective function acceptable for convergence is set to 1e-6). The objective function approached zero, indicating a highly accurate solution in matching the joint distributions of both household- and individual-level attributes. We parallelly generated subpopulations of multiple spatial units on a high-performance computing environment with 800 computing nodes, which took approximately 30 minutes to complete the optimization.

### 3.7 Model validation

#### 3.7.1 Marginal distribution at subzone-level

We performed the optimization for multiple subzones in Shenzhen, thereby generating a synthetic population with geographic spatial area identifiers. The same motif pool extracted from the household survey was used for each optimization process, whereas the marginal distribution of age varied among the subzones. As the demographic data used do not include gender and household size distributions at the subzone level, we use the marginal at the city level to represent the distribution of each subzone. Neglecting the spatial heterogeneity of these attributes can be resolved if the relevant data are available. Therefore, the population synthesizer is expected to generate multiple subpopulations such that each subpopulation can satisfy the age distribution of the subzones, while the entire population can satisfy the distribution of household size, gender, and other high-dimensional attributes at the scale of the whole city.

**Fig 4:**
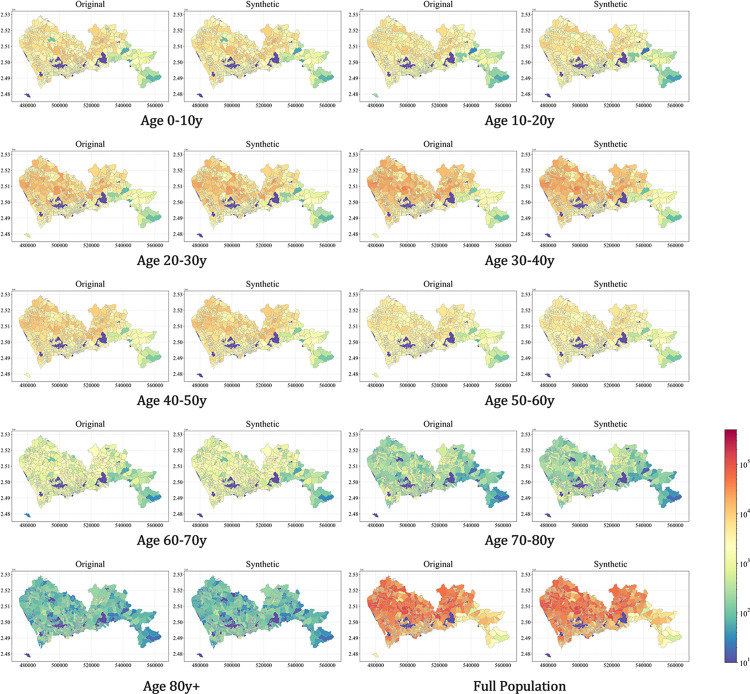
Spatial distribution for full population by age group across subregions. **The basemap shapefile can be accessed at** *https*:*//www*.*kaggle*.*com/datasets/keminzhu/basemap-shenzhen-subzones*.

To assess the accuracy of the generated synthetic population, we compared the number of people of each synthetic subpopulation with the true ground value of the marginal distribution in the demographic data. Fig 4 shows the synthsis results and spatial distribution of different age groups and the full population in the real data, compared on the same scale. It can be seen that for each group of original and synthetic populations, their spatial hot spot is highly consistent, indicating that the proposed model can accurately reflect the spatial distribution characteristics of the population.

**Fig 5:**
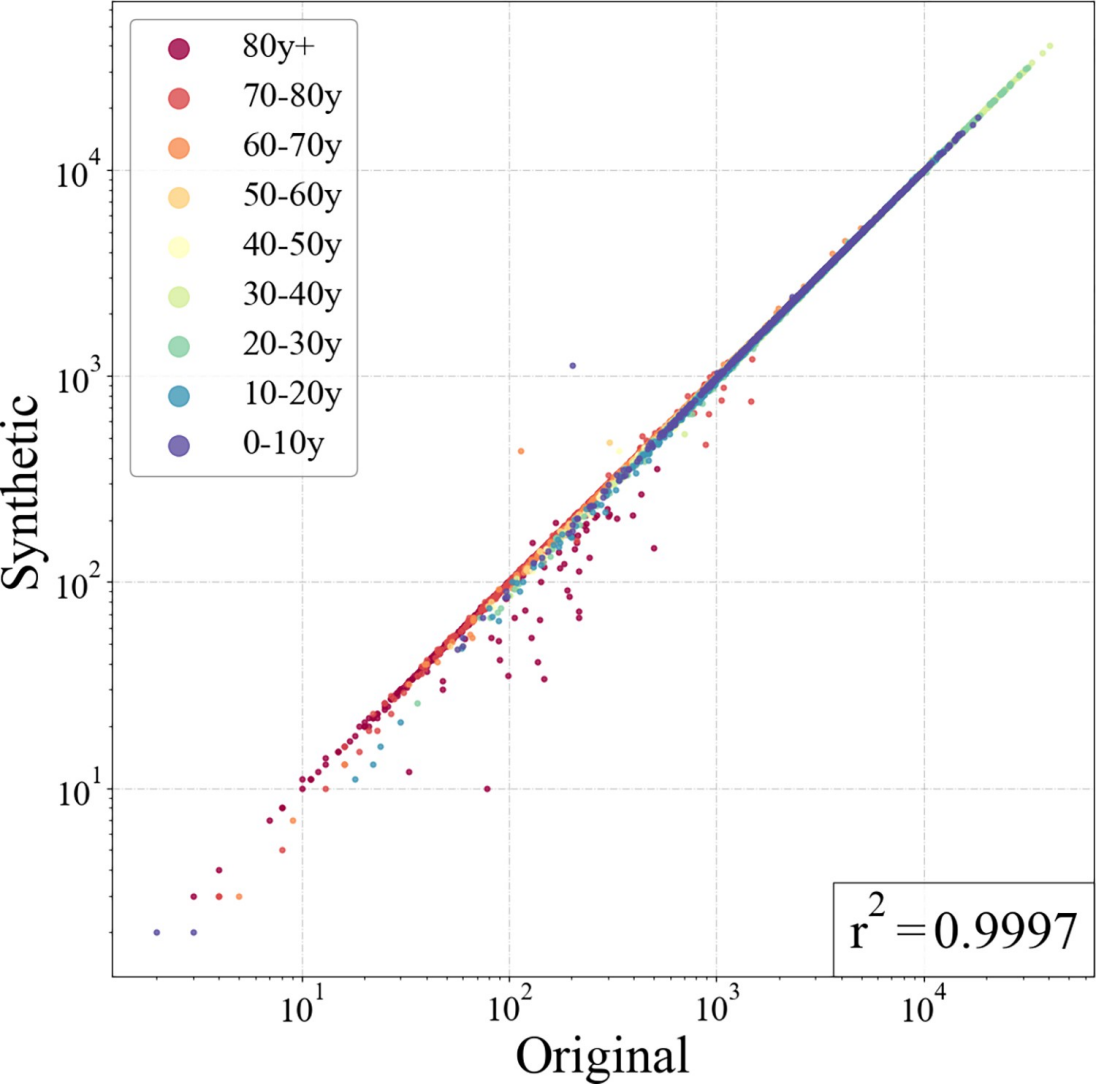
Age distributions obtained from the demographic data and synthetic population at the subzone level, where each point represents the number of people in a certain age group within a subzone.

The quantified analysis results of this comparison are presented in Fig 5, where each point represents the number of individuals living in a certain community within a particular age group, with different age groups distinguished by color. The generated synthetic population is highly consistent with the true values in terms of age distribution, with an r-squared reaching over 0.99, with some of the relatively larger deviations concentrated in the lower left side. Most of them are people over 70 years old, which is mainly due to the highly youthful demographic structure of the study city, with a small number of elderly people in some communities, leading to large relative errors. Overall, the generated synthetic population accurately reflected the spatial distribution of the population in the study area, and the proposed model further exhibited high robustness at the subgroup level.

#### 3.7.2 Marginal/joint distribution at the city level

**Fig 6:**
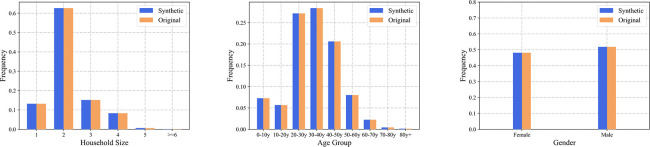
Comparison of marginal distributions obtained from the demographic data and synthetic population for household size, age, and gender.

In this section, we further examine the consistency between the generated synthetic population and real data in the marginal and joint distributions of the attributes. The main attributes examined include household size, age distribution, and gender distribution. As marginal data for gender and household structure are only available at the city level, this part of the test was performed at the level of the whole city population. The results of the comparison are presented separately in Fig 6, for which the marginal distributions of the overall synthetic population were obtained by summing over the subpopulations. The model fits the 1-D marginal distributions well, with errors in the distributions for each attribute within 0.01%, with the largest error observed for the proportion of households with a size of 2, which is 2.23*10^−5^.

**Fig 7:**
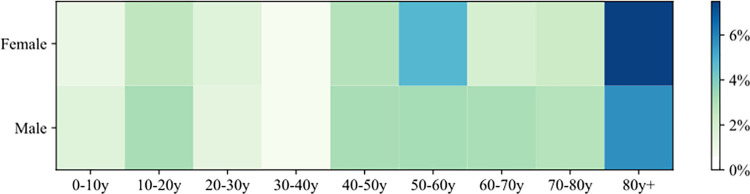
Comparison of the joint distribution of age-gender obtained from the survey dataset and synthetic population.

To further quantify the model consistency, we compared the 2-dimensional (age and sex) frequencies from the generated synthetic population to those computed from the survey data. Due to the absence of subzone-level joint distribution data at the subzone level, this comparison was performed at the level of the entire population. Fig 7 illustrates the comparison results between the survey data and the synthetic population. It focuses on the joint distribution of age-gender groups. The color-coding within each cell signifies the proportion of the population within a specific group. Notably, the largest absolute deviation in the distribution occurs in the (40-50y, female) group. However, this discrepancy is accentuated by the lower representation of the older age group in the overall population, resulting in a relatively higher relative error. The greatest relative error is observed in the 80y+ female group, accounting for approximately 7.6%.

#### 3.7.3 Distribution of household motifs

As mentioned previously, one of the technical difficulties in existing population synthesis methods is capturing and reproducing the interdependency between agents within the same household. Particularly for individual-level epidemic modeling, the age structure of family members may influence the subsequent transmission simulation process. Herein, we compared the different methods by examining the differences between the distributions of household motifs in the simulated results and survey data.

**Fig 8:**
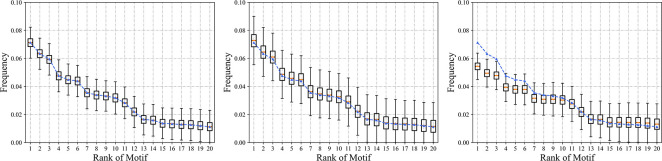
Comparison of the motif distribution in the synthetic populations generated by different methods, with motifs ordered according to the survey data.

Fig 8 shows the ability of the population synthesizer to capture the distribution of household structures. The rank of the motifs was obtained from the distribution of household structures in the survey data. In the absence of observations of the entire population, we assumed that the survey data are sufficiently representative and used the distribution of household structures as the ground truth for testing the model, which is represented by the dashed line. The boxplot represents the frequency of motifs obtained from the synthetic population in repeated experiments for the proposed MHO method and the two benchmark methods. Among the three population synthesizers, MHO and DI showed a superior ability to preserve the distributional properties of motifs, whereas the frequencies of the first 11 motifs simulated by IPF were exclusively lower than the observation. This systematic underestimation was caused by the failure to reproduce the dominance of minority household structures in the population. The mean values of the motif distributions obtained from the DI method simulations in multiple experiments were consistent with observations. This is due to the direct replication of existing households without adjusting the weights, which eventually approaches the distribution of observations after multiple experiments. However, the variances in the simulated distribution of the DI were significantly higher than those of the MHO because the performance of the DI was highly dependent on the quality of the survey data. Bias may be introduced when survey data are not sufficiently representative or have a small sample size. In contrast, the MHO method can better preserve the distribution properties of household structures and has the potential to capture within-household interdependency.

#### 3.7.4 Within-household interdependency

This section assesses the ability of the proposed model to capture the cross-age interdependence of household members, which we describe by constructing a contact matrix. For agents within the same household in the synthetic population, a pair of ages is recorded in the corresponding row and column of the matrix. For example, a household coded as "*(M*,*3)+(F*,*3)+(F*,*0)*" contains two adults in their thirties and a child under ten. In this case, associations between 30-40y and 0-10y were counted twice, while 30-40y and 30-40y were counted once.

**Fig 9:**
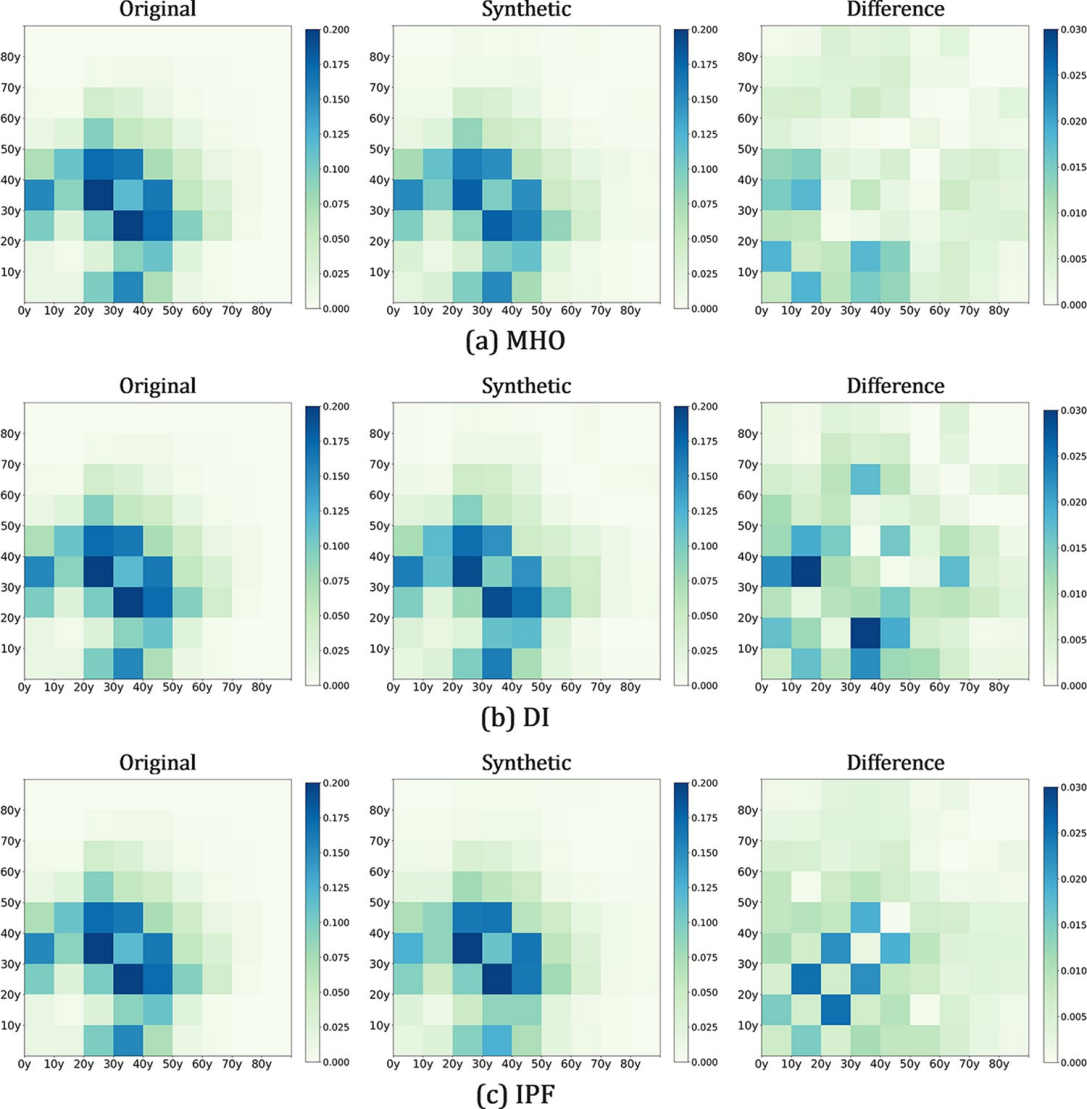
Comparison of interdependency distributions in simulated populations using different methods with those in the survey data, where the value of each cell represents the average frequency of the corresponding cross-age relationship in households.

Fig 9 illustrates the household contact matrix for the synthetic population. To ensure the stability of the results, the matrix of the populations generated using the same method in repeated experiments was averaged. The three synthesizers captured and reproduced the structural features of the original contact matrix to some extent. Among the synthesizers, MHO achieved the best simulation effect, with a sum of mean absolute error of 0.40 less than those of DI (0.58) and IPF (0.48). The distribution of errors was not uniform, with IPF errors concentrated in adjacent age groups, while DI’s largest deviations were in the 2nd and 4th age groups, at 0.018.

### 3.8 Impact on disease transmission

#### 3.8.1 Agent-based epidemic model overview

**Fig 10:**
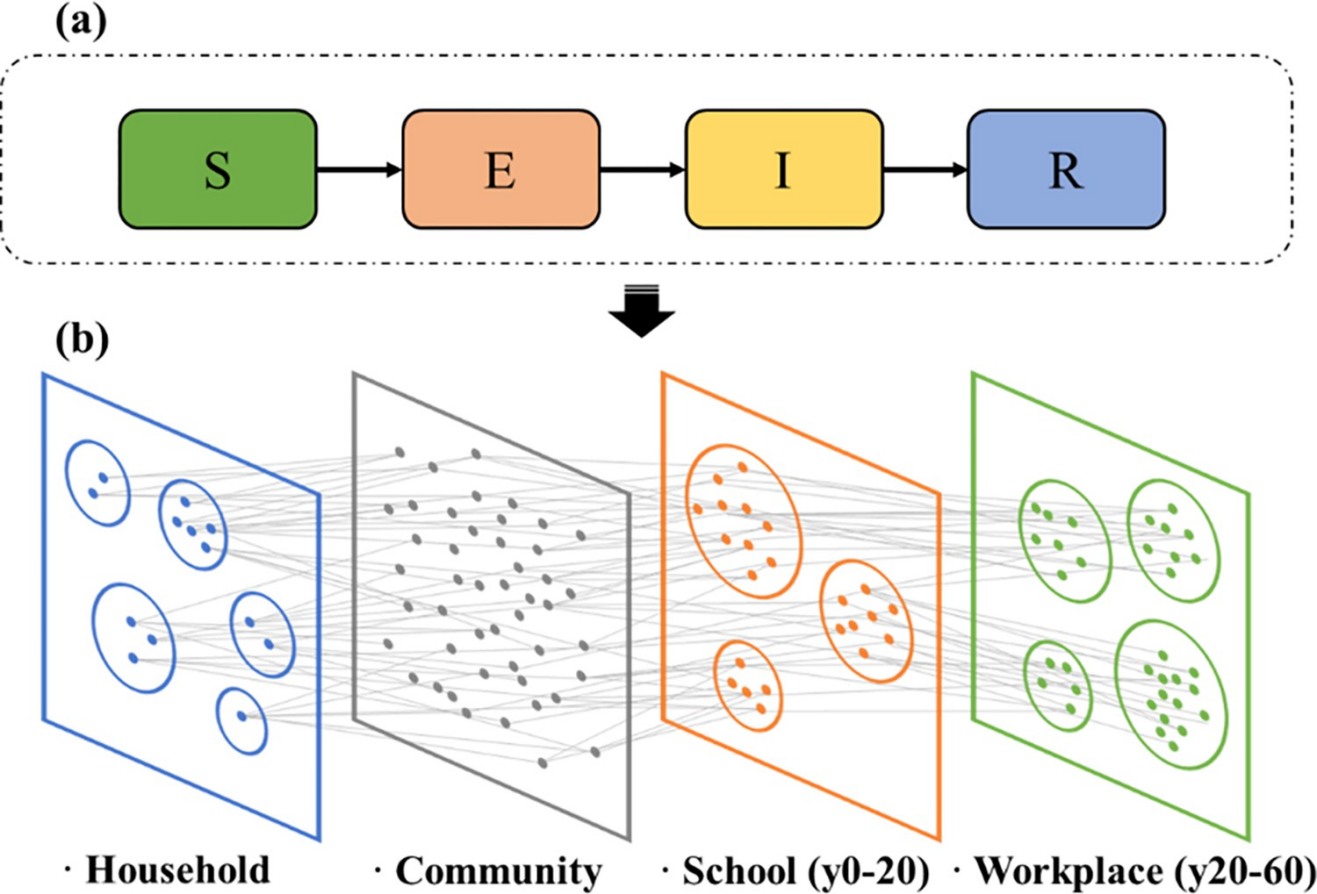
Framework of the agent-based epidemic model. (a) The compartmental model used to describe the natural history of the infectious disease between the states. (b) Schematic illustration of the weighted multilayer contact network. Details of the epidemic model and the transitions between compartments are provided in the S1 Text.

In this section, a stochastic, discrete-time, agent-based model was constructed to further evaluate the impact of the population synthesis approach on the follow-up epidemic transmission process. This model was modified from [67]). Specifically, in Section 4.3, we consider a transmission model in which the pathogen spreads on synthetic weighted contact networks inferred from populations generated by the MHO, DI, and IPF methods outlined in *4*.*3*. As shown in Fig 10, the contact networks consisted of four layers representing the network of interactions among agents in the following settings: (1) household, (2) workplace, (3) schools, and (4) community. Connections between the two agents in the household layer were inferred from the household profiles of synthetic populations. The work and school layers only included agents aged 0–20 and 20–60 years old respectively, whereas the household and community layers included agents of all ages. For simplicity, employment and enrollment rates are not considered in the model; each agent aged 0–60 years represents a node in the workplace or school layer. The transmission probability per contact depends on the contact type. These values correspond to relative weightings of 10:2:2:1 (Kerr et al., 2021) [68] (i.e., households, schools, workplaces, and community contacts), chosen for consistency with both time-use surveys (Lader, Short, & Gershuny, 2006) [69] and studies of infections with known contact types (Zhang et al., 2020) [70].

The natural history of the disease is captured as a simple *Susceptible-Exposed-Infectious-Recovered* model. The model assumes that susceptible individuals (S) are exposed to the disease through contact with infected individuals, and subsequently transition to exposed compartments (E), where they are infected but not yet infectious. Symptomatic individuals were assigned an incubation period of 2 days to manifest symptoms (I). After the onset of symptoms, the infectious agents remain infectious for another 10 days and recover (R), gaining permanent immunity against further infection.

#### 3.8.2 Simulation of epidemic transmission

In this section, we analyze the transmission simulation results for the synthetic populations. For each population generated by MHO, DI, and IPF in Section 4.3, multi-layer contact networks and individual models were constructed according to the settings described above. Specifically, the model was initialized with 100 randomly selected infected seeds from the ages 0-20y and their compartments were set to *Exposed*. The infected agents in the simulation were recorded each day until the maximum number of simulation days (80 days) was reached.

**Fig 11:**
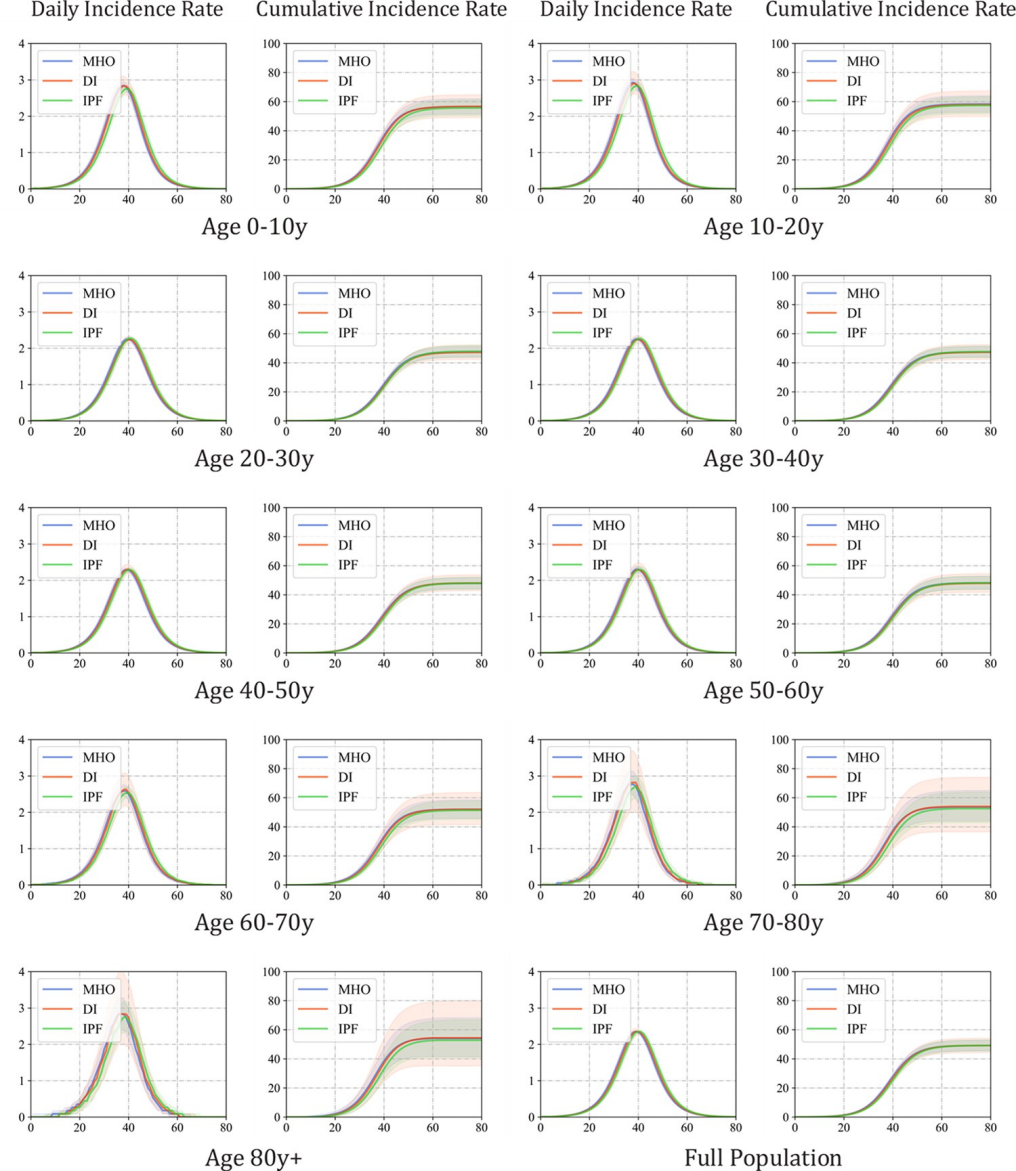
Epidemic curve simulated with a different synthetic population.

The simulated epidemic curves are given in Fig 11, where the daily incident rate represents the number of new cases (*Susceptible*→*Exposed*) within a day as a proportion of the number of their age group or the whole population. Herein, we focused on metrics, including the peak incidence rate, peak date, and attack rate, which are the most critical indicators of concern in real-world epidemic prevention and control. From the full age-epidemic curve, the results of transmission processes on three types of synthetic populations did not show significant differences in the mean values of attack rate and peak incidence rate, while the epidemic curve of the DI population showed greater uncertainty, with the standard variance of full age-final attack rate and peak incidence rate in 1000 repeat experiments being 0.99 and 0.10, respectively, which are much higher than those of MHO (0.12 and 0.03) and IPF (0.12 and 0.03). This is consistent with the prior analysis of the household motif distribution in Fig 8, which indicated that the DI method is more sensitive to the representation of sampled survey data used for synthetic population generation; thus, the population is less stable in terms of transmission characteristics.

Among all three types of synthetic populations, the infection peak date of age group y0-20 arrived earlier than the others, as the initial seed was set in a school, making agents in these age groups more likely to be infected in the early stage. However, for the IPF population, both age-specific and full-age-peak date arrives slightly later than for the other two types of populations. In the age groups of y60+, the difference in peak time will be more significant, with the peak time simulated by the IPF method lagging behind that of the MHO method by two days. We speculate that this difference is due to the distribution of household motifs in synthetic populations. Additionally, although the mean value of the peak date of the DI population was the same as that of the MHO population, the uncertainty was significantly greater according to the percentile interval width.

In conclusion, simulating disease transmission based on different synthetic populations with consistent marginal attribute distributions does not result in significant differences in the attack rate at the end of the simulation, whether it is for a specific age group or the full population. However, it does have an impact on the daily incident rate during the epidemic and the timing of the epidemic peak. We speculate that this is because the final infection size in the population at equilibrium is mainly influenced by the marginal attribute distribution, and there is no significant difference between different synthetic population generation methods. However, the proportion of household structures in different synthetic populations affects the dynamics of disease transmission dynamic in epidemic simulations. Therefore, accurately modeling the distribution of household structures is necessary for precise analysis and prevention and control in real-world epidemic modeling.

## 4. Discussion

In this research, we introduced a heuristic optimization-driven approach to craft synthetic populations tailored for infectious disease transmission dynamics. By weaving in insights from typical household structures and their distribution patterns sourced from survey data, we bolstered the synthesizer’s capability to articulate the structural relationships within households. Our emphasis was directed towards formulating populations underpinned by dependable contact dynamics. This study’s pivotal contributions encompass as followed:

First, based on an analysis of household survey data from multiple countries and regions, we found that household structures in real-world populations exhibit significant power-law distribution characteristics, such that a limited number of household structure types are sufficient to represent the entire population with adequate representativeness.

Second, we proposed an MHO population synthesis method to enhance the reliability of simulating household contact relationships by incorporating typical household structure information supporting agent-based modeling of infectious diseases. The performance of proposed method was evaluated by generating synthesized populations at the subzone-level of over 17 million people in Shenzhen, China. Generated synthetic populations’ marginal attributes, age-gender combinations, and household structure distributions were compared with demographic and survey data

Finally, further analysis of the impact of different population synthesis methods on household contact relationships and the transmission of infectious diseases using an agent-based *SEIR* disease transmission model with a multilayer contact network constructed from synthetic populations showed that, even with the same input data, different population synthesis methods can cause differences in peak dates and peak incidence rates in epidemic simulations. In the broader context of this field, previous studies [71–73] have explored the role of household and demographic structure in disease transmission, providing a foundational understanding that informs our approach.

While this study primarily focused on age-related factors due to their significant influence on disease dynamics and the availability of relevant data, our methodology possesses the adaptability to be extended to other attributes, such as immunity, when corresponding data becomes accessible. This flexibility opens up exciting avenues for future research, enabling the generation of synthetic populations structured by diverse factors that impact disease transmission.

Futher research is required to overcome the limitations, e.g., better handling high-dimensional attributes. The proposed approach relies on a small number of household structures to represent the population, which means that, when dealing with population synthesis problems that involve more attributes of interest beyond age and gender, the number of required motifs increases significantly. Reducing computational burden can be achieved by addressing this issue. It is also important to note that our model currently does not incorporate factors like income, which recent studies suggest may affect disease transmission dynamics. This limitation is an area for potential future refinement. Furthermore, testing on a wider range of datasets is required to evaluate the computational performance of the method when dealing with populations that contain a greater variety of household structures.

## Supporting information

S1 Data Excel spreadsheet containing, in separate sheets, the underlying numerical data for Figs 2, 3, 4, 5, 6, 7, 8, 9, and 11. The numerical data for Fig 10 is stored in a shapefile, which can be accessed through this link: https://www.kaggle.com/datasets/keminzhu/basemap-shenzhen-subzones.
(XLSX)

S1 Text Information on the process of generating the synthetic population network and the epidemic model. This passage provides a detailed description of the process involved in generating the multi-layered contact network *G* based on synthetic population in the model. It includes information about the age groups of agents targeted by each layer and the configuration of weight. **Fig A. Parameters used in the infectious disease model**. Encompasses all parameter values used in our *S-E-I-R* model, along with their descriptions.
(DOCX)

S2 Text The power-law distribution test for household structures in other survey datasets. Including tests for other regions in China (CFPS dataset) and other countries worldwide (IPUM dataset). **Fig A in S2 Text.** Including tests for Shanghai, Guangdong, Liaoning, Henan, Gansu, and Other Areas in China datasets, sourced from **http://www.isss.pku.edu.cn/cfps/download**. **Fig B in S2 Text.** Including tests for 15 datasets in different countries from different year, sourced from https://international.ipums.org/international/.
(DOCX)

S1 Table List of top 100 household structures in the transportation survey of Shenzhen. These frequently occurring household structures cover over 88% of the total population in the survey data. The proportions of these household structures are set as the initial guess of the dicision vector for the subsequent combinatorial optimization algorithm.
(DOCX)

S2 Table Numerical results of epidemic simulation based on different population synthesis methods. The Peak Date, Peak Incidence Rate, and Attack Rate of the epidemic curve, including both the entire population and various age groups, are provided. Standard deviations (SD) and confidence intervals (CI) are also included.
(DOCX)
