# Supplementary material for: Generating synthetic population for simulating the spatiotemporal dynamics of epidemics
Source: PLoS Comput Biol. 2024 Feb 12;20(2):e1011810. doi: 10.1371/journal.pcbi.1011810 (PMC10890746; doi:10.1371/journal.pcbi.1011810)
Supplement: S1 Text — This passage provides a detailed description of the process involved in generating the multi-layered contact network G based on synthetic population in the model. It includes information about the age groups of agents targeted by each layer and the configuration of weight. Fig A. Parameters used in the infectious disease model. Encompasses all parameter values used in our S-E-I-R model, along with their descriptions. (DOCX) [file pcbi.1011810.s002.docx]

**S1 Text 1. Information on the process of generating the synthetic population network and the epidemic model**

# Network generation

In this study, we generated a multi-layered contact network $G= \{g_{L}(V_{L},E_{L})|L\in(L_{H},L_{W},L_{S},L_{C})\}$ among agents based on the synthetic population generated by the proposed MHO and control methods (DI, IPF). This network serves as the foundation for subsequent disease transmission simulations. $V_{L}$ represents the agents involved in the network, while $E_{L}$ denotes the contacts among agents that carry the risk of disease transmission. Different types of contacts are assigned heterogeneous transmission risks, and the transmission probability weights of contact on $g_{L}$ is expressed as $w_{L}$.

- Household Layer ($L_{H}$): The contact network within the household layer was strictly generated according to the household structure in the synthetic population. Specifically, agents in the synthetic population were grouped by their household identification (*HID*) field, and members within the same household were connected to each other. The $L_{H}$ was static, meaning that individual household contacts did not change during each simulated day.
- Workplace Layer ($L_{W}$): The workplace contact network includes agents in age group y20-60, and all agents on this network are randomly divided into multiple subpopulations representing different workplaces. The contacts on the $L_{W}$ is relatively fixed, meaning that on each simulation day, each agent will encounter a certain number of others within their respective workplaces.
- School Layer ($L_{S}$): The setting of the school contact network is similar to that of the workplace network, but the agents included in $L_{S}$ are all in the age group of y0-20.
- Community Layer ($L_{C}$): The community network describes the contacts between agents during their leisure activities. We consider agents in a spatial unit in the synthetic population as a group and each agent randomly contacts others in the same unit on each simulation day. $L_{C}$ includes agents of all age groups.

According to ([Kerr et al., 2021](#_ENREF_22)) [68], the relative risks weights $w_{L}$ of $L_{H},L_{W},L_{S},L_{C}$ were set as 10:2:2:1, indicating that the risk of transmission is highest within households and relatively lower in community settings.

# Agent-based epidemic model

To analyze the impact of the population synthesizer on disease transmission simulation, we developed a stochastic discrete agent-based infectious disease model. Each agent in the synthetic population was assigned a health status attribute (i.e., compartment), which included Susceptible, Exposed, Infectious, and Recovered states.

At model initialization, all agents were set to Susceptible. Then, $n_{seed}$ agents in the age group y0-20 were randomly selected as initial infection seeds, and their compartment was reset to $Exposed$. Compartment transitions occurred in the form of $Exposed$-$Infectious$-$Recoverd$, with transition intervals following a log-normal distribution. In each simulation day, infectious agents had a probability $p$ to infect other agents they encountered.

$$p=pTrans*w_{L}$$

Where $pTrans$ represents the probability of transmission events occurring during daily contacts, while $w_{L}$ denotes the weight coefficients of different layers of network ([Yin et al., 2021](#_ENREF_43)) [67]. The parameter settings of the model are shown in the following table.

**Table A.** **Parameters used in the infectious disease model**

| **Parameter** | **Value** | **Description** |
| --- | --- | --- |
| $n_{seed}$ | 100 | Number of initial infectious agents |
| $pTrans$ | 0.05 | Probability of transmission during contact |
| $w_{H}$, $w_{W}$, $w_{S}$, $w_{C}$ | 10,2,2,1 | Relative transmission risks weights of networks (household/workplace/school/community) |
| $\mu_{EI}$ | 2 | Mean of the lognormal distribution for Exposed-to-Infectious transition |
| $\mu_{IR}$ | 10 | Mean of the lognormal distribution for Infectious -to-Recovered transition |
| $\sigma_{EI}$ | 0.1 | Standard deviation of the lognormal distribution for Exposed-to-Infectious transition |
| $\sigma_{IR}$ | 0.1 | Standard deviation of the lognormal distribution for Infectious -to-Recovered transition |
| $n_{iter}$ | 80 | Simulation time (days) |
| $n_{repeat}$ | 1000 | Number of repeat experiments |
