# Supplementary material for: Generating synthetic population for simulating the spatiotemporal dynamics of epidemics
Source: PLoS Comput Biol. 2024 Feb 12;20(2):e1011810. doi: 10.1371/journal.pcbi.1011810 (PMC10890746; doi:10.1371/journal.pcbi.1011810)
Supplement: S2 Text — Including tests for other regions in China (CFPS dataset) and other countries worldwide (IPUM dataset). Fig A in S2 Text. Including tests for Shanghai, Guangdong, Liaoning, Henan, Gansu, and Other Areas in China datasets, sourced from http://www.isss.pku.edu.cn/cfps/download. Fig B in S2 Text. Including tests for 15 datasets in different countries from different year, sourced from https://international.ipums.org/international/. (DOCX) [file pcbi.1011810.s003.docx]

To verify the applicability of the power-law distribution characteristics of household structure in different geographic regions and scales as found in this study, we further fit the data using the logarithmic regression method in other datasets.

Fig A shows the fitted results of household structure distribution in six datasets, including five provinces in China and other regions, obtained from the household survey conducted since 2018 by the China Family Panel Studies (CFPS). According to the logarithmic fitting results, the frequency of household structure in each dataset exhibits power-law distribution characteristics, with regression coefficients ranging from 0.47 to 0.82. The fitting goodness of Guangdong Province data is the lowest ($r^{2}$ = 0.778), while the r-squared values of the other datasets are all above 0.85.

| 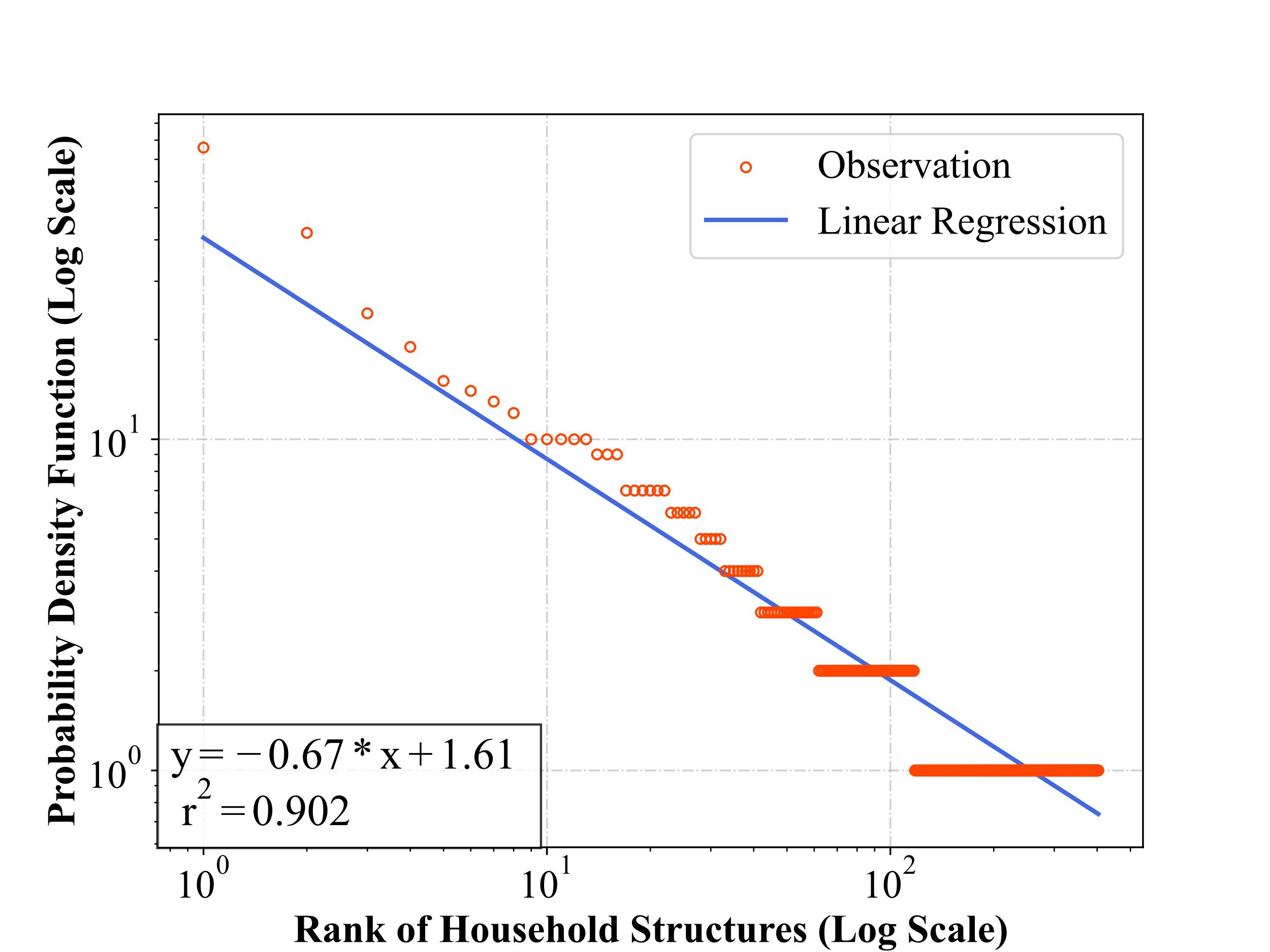 | 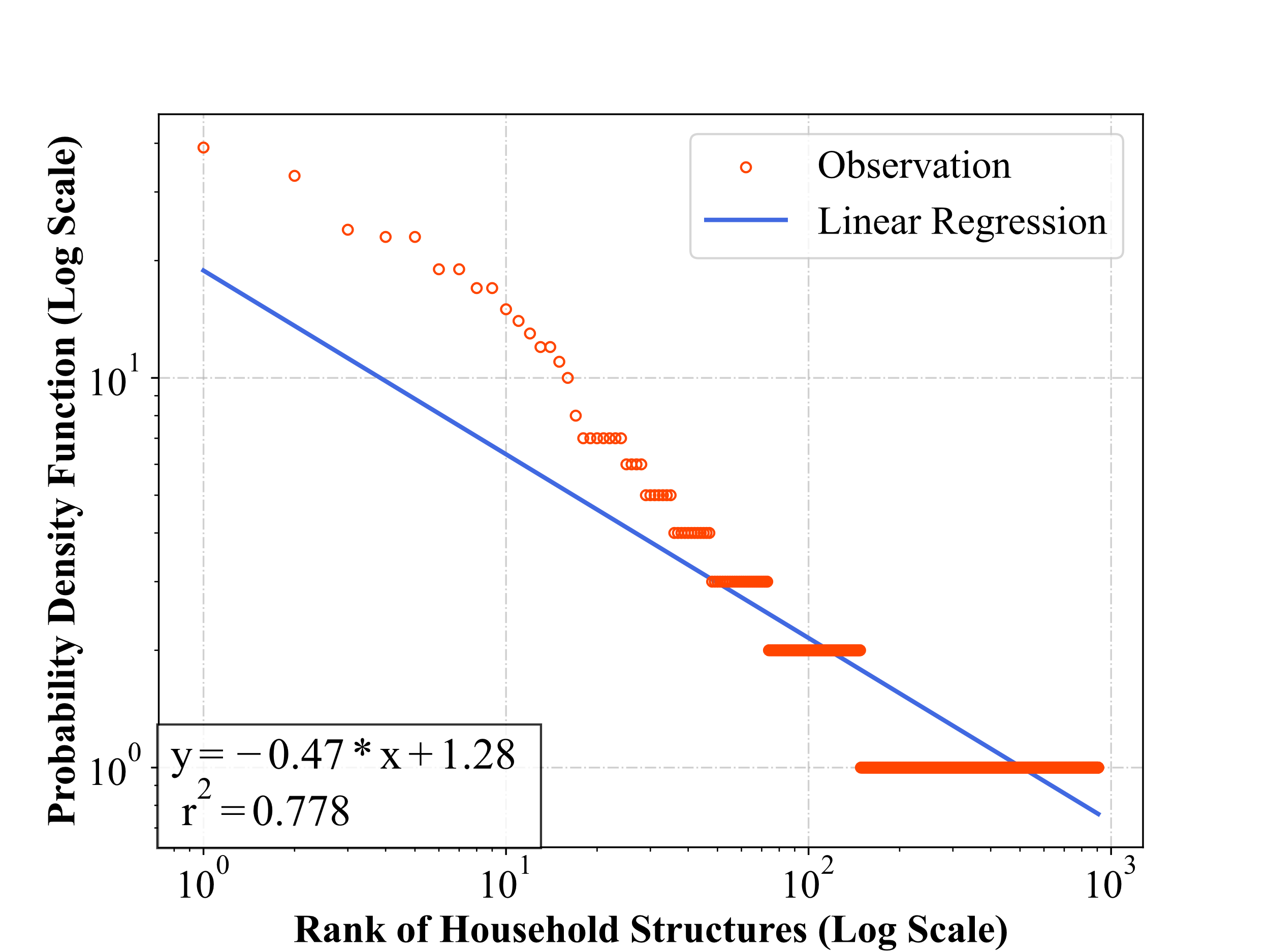 | 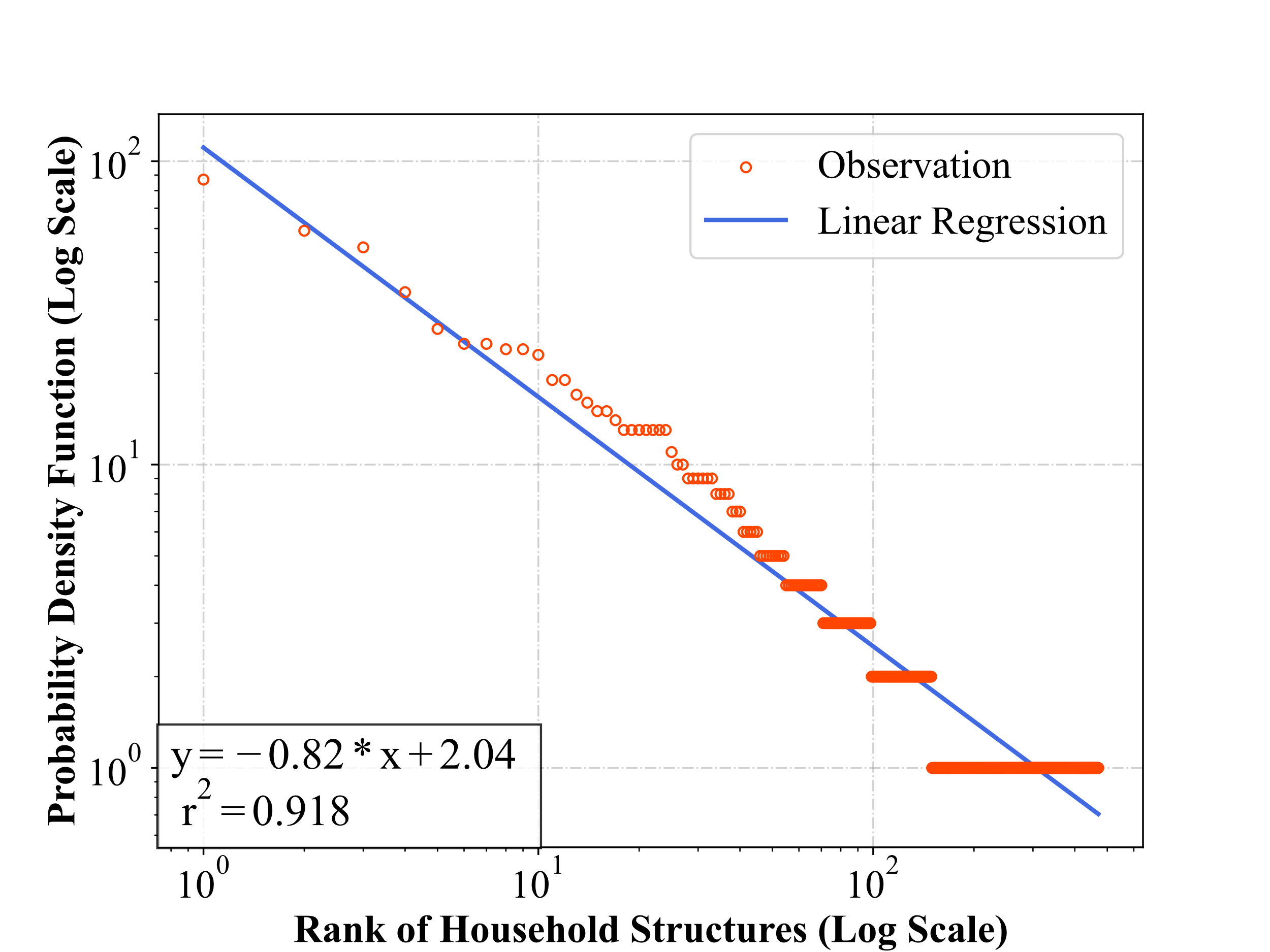 |
| --- | --- | --- |
| Shanghai | Guangdong | Liaoning |
| 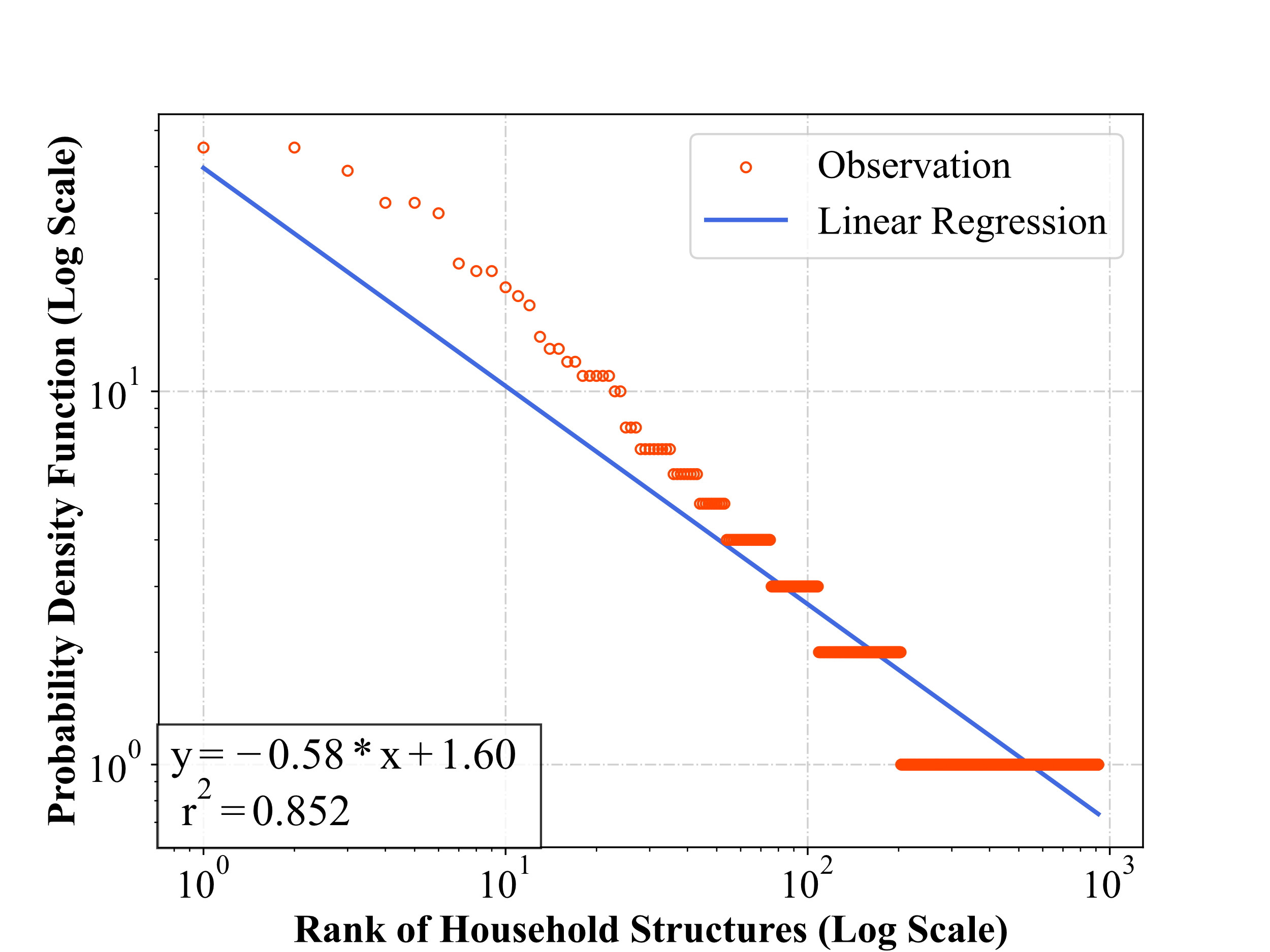 | 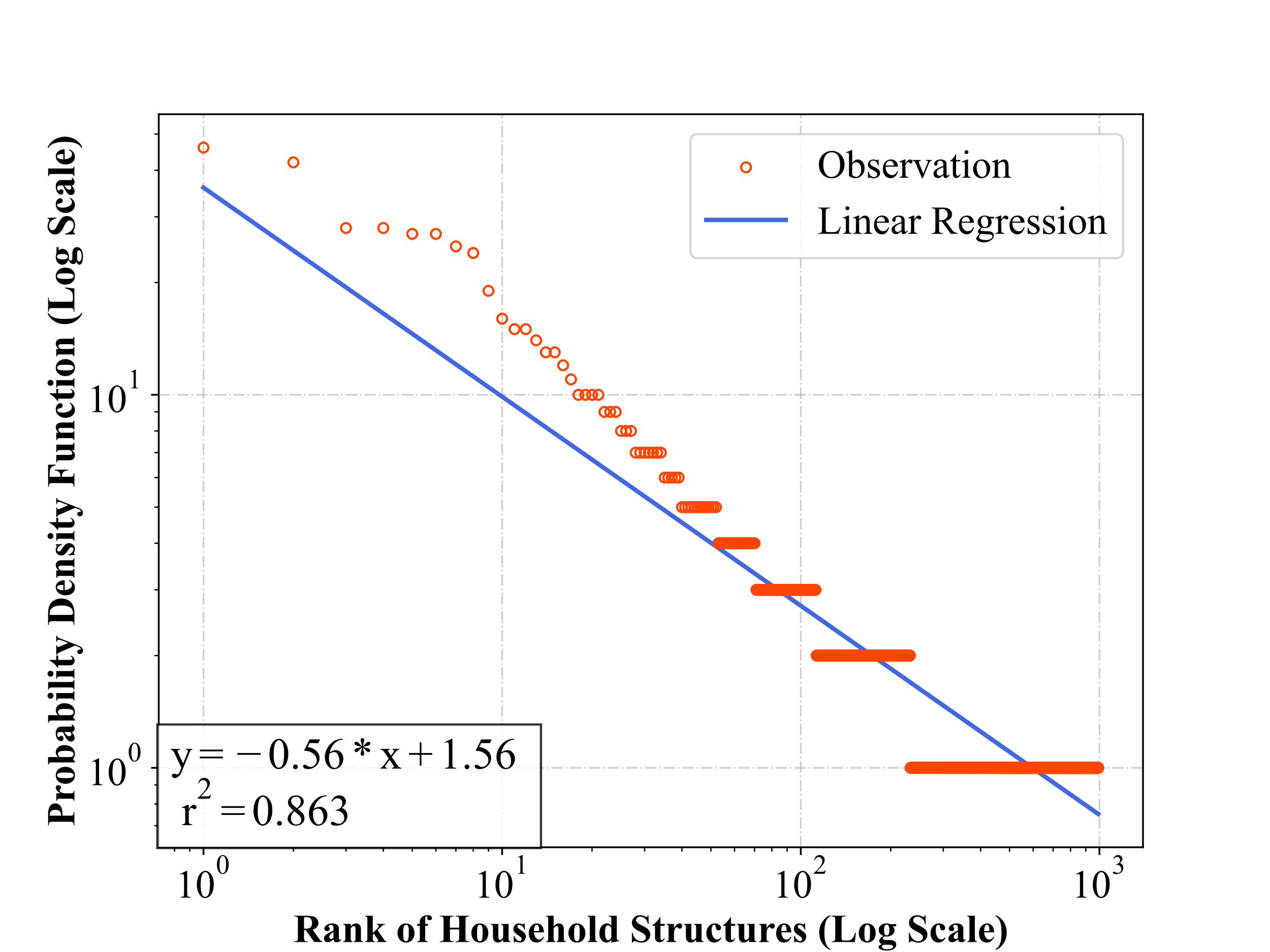 | 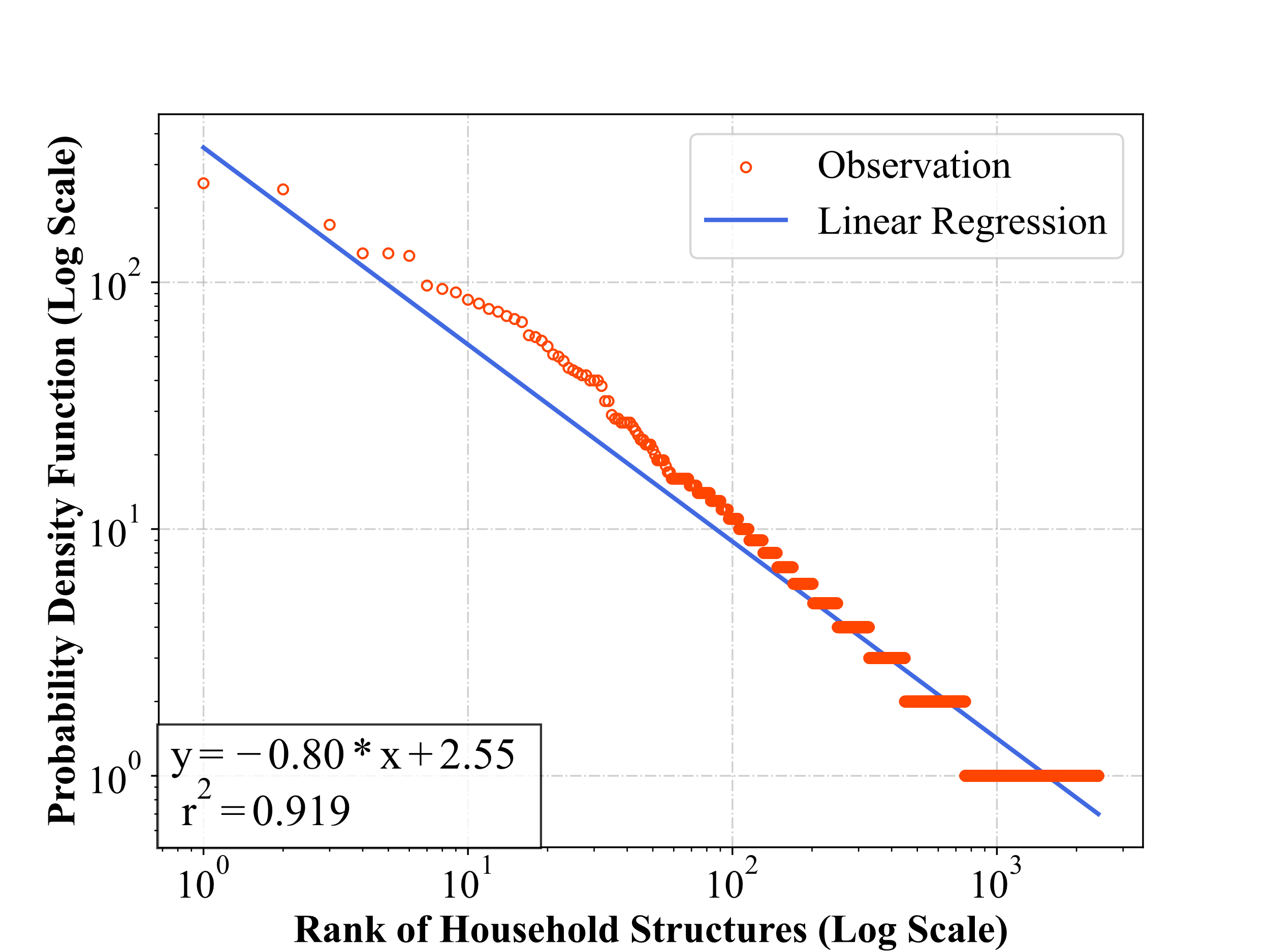 |
| Henan | Gansu | Other Area in China |
| **Fig A The distribution of household structures and regression curve in the CFPS.** | | |

*The data can be obtained by applying at http://www.isss.pku.edu.cn/cfps/download

Fig B shows the fitting results of household structure distribution obtained from survey data of different years in 15 countries other than China, obtained from the Integrated Public Use Microdata Series (IPUMS). It is evident that compared to the CFPS dataset, the power-law distribution characteristics of household structure in the IPUMS datasets are more pronounced, with the lowest $r^{2}$ value being 0.927 (Nepal-2011). The regression coefficients range from 0.99 to 1.63, indicating that the slopes of the regression lines are higher than those of the CFPS dataset, suggesting that a small proportion of family structures are more representative of the entire population. We speculate that this power-law distribution relationship may be universal and more stable in larger geographical areas and populations size.

| 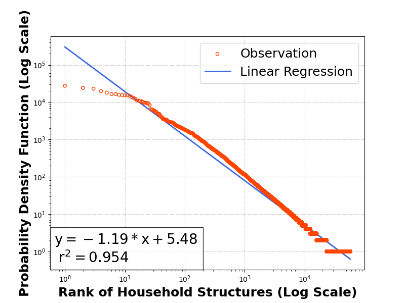 | 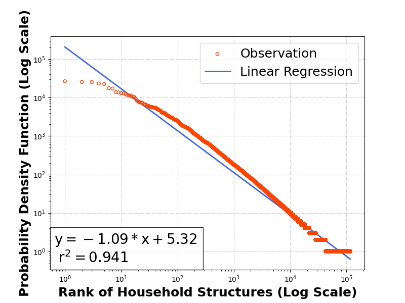 | 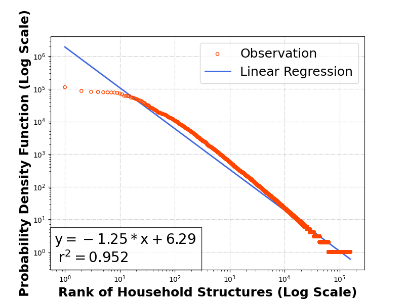 |
| --- | --- | --- |
| Argentina-2010 | Bangladesh-2011 | Brazil-2010 |
| 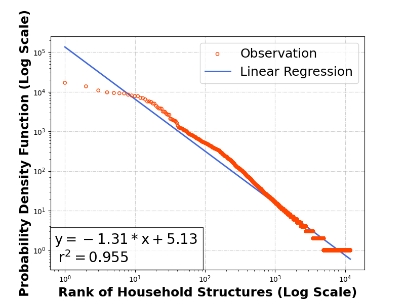 | 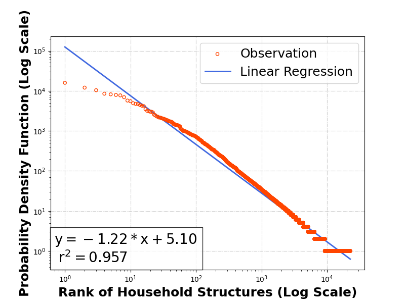 | 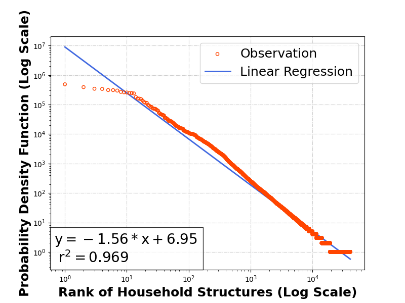 |
| Canada-2011 | Cuba-2012 | France-2011 |
| 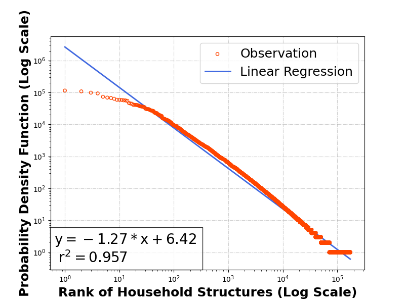 | 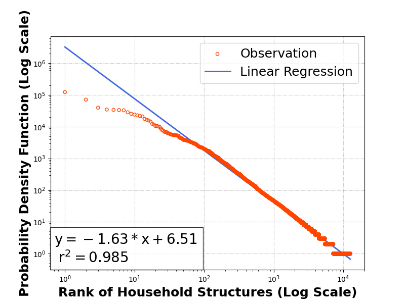 | 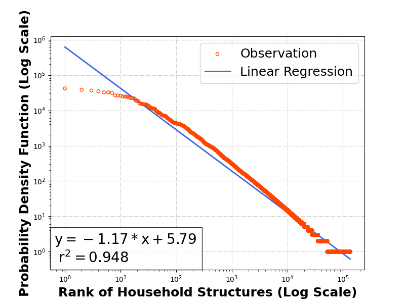 |
| Indonesia-2010 | Italy-2011 | Mexico-2015 |
| 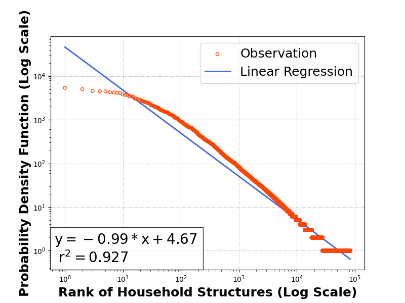 | 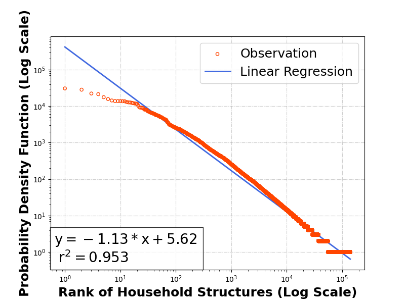 | 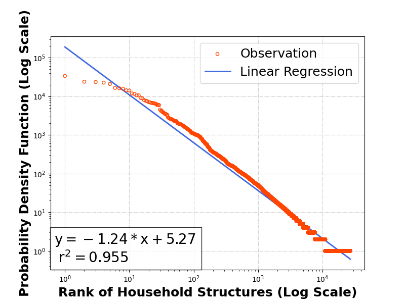 |
| Nepal-2011 | Philippines-2010 | Romania-2011 |
| 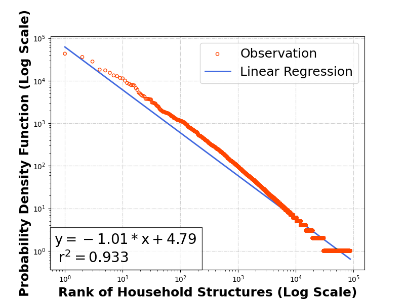 | 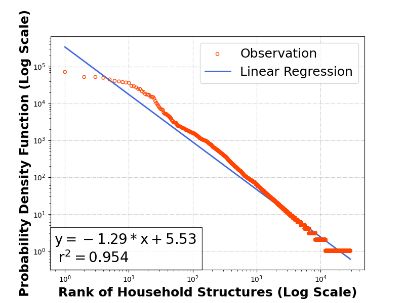 | 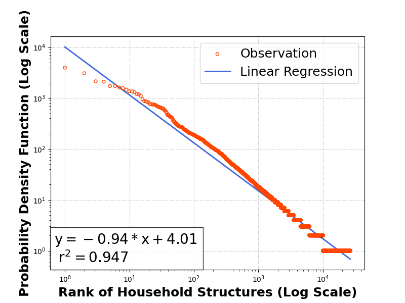 |
| South Africa-2016 | United States-2015 | Zimbabwe-2012 |
| **Fig B The distribution of household structures and regression curve in the IPUMS.** | | |

*The data can be obtained by applying at https://international.ipums.org/international/
