# Supplementary material for: Generating synthetic population for simulating the spatiotemporal dynamics of epidemics
Source: PLoS Comput Biol. 2024 Feb 12;20(2):e1011810. doi: 10.1371/journal.pcbi.1011810 (PMC10890746; doi:10.1371/journal.pcbi.1011810)
Supplement: S1 Table — These frequently occurring household structures cover over 88% of the total population in the survey data. The proportions of these household structures are set as the initial guess of the dicision vector for the subsequent combinatorial optimization algorithm. (DOCX) [file pcbi.1011810.s004.docx]

**Table S1. List of top 100 household structures in the transportation survey of Shenzhen**

| **rank** | **h_code** | **x_init** | **h_count** | **h_label** |
| --- | --- | --- | --- | --- |
| 1 | (((0, 2), 1), ((1, 3), 1)) | 7.13% | 2118 | female(20-30) male(30-40) |
| 2 | (((0, 2), 1), ((1, 2), 1)) | 6.33% | 1880 | female(20-30) male(20-30) |
| 3 | (((0, 3), 1), ((1, 3), 1)) | 5.96% | 1769 | female(30-40) male(30-40) |
| 4 | (((0, 3), 1), ((1, 2), 1)) | 4.76% | 1413 | female(30-40) male(20-30) |
| 5 | (((0, 3), 1), ((1, 4), 1)) | 4.48% | 1332 | female(30-40) male(40-50) |
| 6 | (((0, 2), 1), ((1, 4), 1)) | 4.39% | 1303 | female(20-30) male(40-50) |
| 7 | (((0, 4), 1), ((1, 3), 1)) | 3.55% | 1054 | female(40-50) male(30-40) |
| 8 | (((1, 3), 1),) | 3.40% | 1009 | male(30-40) |
| 9 | (((1, 2), 1),) | 3.32% | 985 | male(20-30) |
| 10 | (((0, 4), 1), ((1, 4), 1)) | 3.19% | 946 | female(40-50) male(40-50) |
| 11 | (((0, 4), 1), ((1, 2), 1)) | 2.83% | 840 | female(40-50) male(20-30) |
| 12 | (((0, 2), 1),) | 2.19% | 651 | female(20-30) |
| 13 | (((0, 3), 1),) | 1.65% | 490 | female(30-40) |
| 14 | (((1, 4), 1),) | 1.59% | 472 | male(40-50) |
| 15 | (((1, 2), 1), ((1, 5), 1)) | 1.37% | 407 | male(20-30) male(50-60) |
| 16 | (((0, 3), 1), ((1, 5), 1)) | 1.33% | 395 | female(30-40) male(50-60) |
| 17 | (((0, 2), 1), ((1, 5), 1)) | 1.29% | 383 | female(20-30) male(50-60) |
| 18 | (((0, 4), 1), ((1, 5), 1)) | 1.27% | 377 | female(40-50) male(50-60) |
| 19 | (((0, 2), 1), ((0, 5), 1)) | 1.20% | 355 | female(20-30) female(50-60) |
| 20 | (((1, 0), 1), ((1, 3), 1)) | 1.12% | 333 | male(0-10) male(30-40) |
| 21 | (((0, 5), 1), ((1, 3), 1)) | 1.00% | 298 | female(50-60) male(30-40) |
| 22 | (((1, 1), 1), ((1, 4), 1)) | 1.00% | 297 | male(10-20) male(40-50) |
| 23 | (((0, 5), 1), ((1, 4), 1)) | 0.99% | 293 | female(50-60) male(40-50) |
| 24 | (((0, 3), 1), ((1, 0), 1), ((1, 3), 1)) | 0.84% | 251 | female(30-40) male(0-10) male(30-40) |
| 25 | (((0, 1), 1), ((0, 4), 1)) | 0.84% | 250 | female(10-20) female(40-50) |
| 26 | (((0, 5), 1), ((1, 5), 1)) | 0.84% | 249 | female(50-60) male(50-60) |
| 27 | (((0, 2), 1), ((1, 0), 1), ((1, 3), 1)) | 0.82% | 243 | female(20-30) male(0-10) male(30-40) |
| 28 | (((0, 2), 1), ((0, 4), 1)) | 0.82% | 243 | female(20-30) female(40-50) |
| 29 | (((0, 5), 1), ((1, 2), 1)) | 0.80% | 239 | female(50-60) male(20-30) |
| 30 | (((0, 4), 1),) | 0.76% | 225 | female(40-50) |
| 31 | (((0, 0), 1), ((0, 3), 1)) | 0.75% | 222 | female(0-10) female(30-40) |
| 32 | (((0, 1), 1), ((0, 3), 1)) | 0.62% | 183 | female(10-20) female(30-40) |
| 33 | (((0, 3), 1), ((1, 1), 1), ((1, 4), 1)) | 0.56% | 166 | female(30-40) male(10-20) male(40-50) |
| 34 | (((1, 1), 1), ((1, 5), 1)) | 0.53% | 157 | male(10-20) male(50-60) |
| 35 | (((1, 1), 1), ((1, 3), 1)) | 0.52% | 154 | male(10-20) male(30-40) |
| 36 | (((0, 0), 1), ((0, 2), 1)) | 0.50% | 149 | female(0-10) female(20-30) |
| 37 | (((1, 2), 1), ((1, 4), 1)) | 0.47% | 141 | male(20-30) male(40-50) |
| 38 | (((0, 0), 1), ((0, 3), 1), ((1, 3), 1)) | 0.46% | 138 | female(0-10) female(30-40) male(30-40) |
| 39 | (((0, 2), 1), ((1, 0), 1), ((1, 2), 1)) | 0.45% | 132 | female(20-30) male(0-10) male(20-30) |
| 40 | (((0, 3), 1), ((1, 1), 1), ((1, 3), 1)) | 0.43% | 127 | female(30-40) male(10-20) male(30-40) |
| 41 | (((1, 0), 1), ((1, 4), 1)) | 0.42% | 124 | male(0-10) male(40-50) |
| 42 | (((0, 4), 1), ((1, 1), 1), ((1, 4), 1)) | 0.39% | 116 | female(40-50) male(10-20) male(40-50) |
| 43 | (((0, 3), 1), ((1, 2), 1), ((1, 6), 1)) | 0.39% | 115 | female(30-40) male(20-30) male(60-70) |
| 44 | (((1, 2), 1), ((1, 6), 1)) | 0.39% | 115 | male(20-30) male(60-70) |
| 45 | (((0, 4), 1), ((1, 0), 1), ((1, 3), 1)) | 0.38% | 113 | female(40-50) male(0-10) male(30-40) |
| 46 | (((0, 2), 1), ((1, 1), 1), ((1, 4), 1)) | 0.37% | 111 | female(20-30) male(10-20) male(40-50) |
| 47 | (((0, 3), 1), ((0, 5), 1)) | 0.37% | 111 | female(30-40) female(50-60) |
| 48 | (((0, 0), 1), ((0, 2), 1), ((1, 3), 1)) | 0.37% | 110 | female(0-10) female(20-30) male(30-40) |
| 49 | (((0, 3), 1), ((1, 0), 1), ((1, 2), 1)) | 0.35% | 104 | female(30-40) male(0-10) male(20-30) |
| 50 | (((0, 2), 1), ((1, 2), 1), ((1, 6), 1)) | 0.34% | 101 | female(20-30) male(20-30) male(60-70) |
| 51 | (((0, 0), 1), ((0, 2), 1), ((1, 2), 1)) | 0.31% | 93 | female(0-10) female(20-30) male(20-30) |
| 52 | (((1, 0), 1), ((1, 2), 1)) | 0.31% | 93 | male(0-10) male(20-30) |
| 53 | (((0, 3), 1), ((1, 0), 1), ((1, 4), 1)) | 0.30% | 91 | female(30-40) male(0-10) male(40-50) |
| 54 | (((0, 1), 1), ((0, 4), 1), ((1, 4), 1)) | 0.30% | 89 | female(10-20) female(40-50) male(40-50) |
| 55 | (((0, 2), 1), ((1, 1), 1), ((1, 3), 1)) | 0.30% | 89 | female(20-30) male(10-20) male(30-40) |
| 56 | (((0, 1), 1), ((0, 3), 1), ((1, 3), 1)) | 0.28% | 83 | female(10-20) female(30-40) male(30-40) |
| 57 | (((0, 1), 1), ((0, 4), 1), ((1, 3), 1)) | 0.27% | 79 | female(10-20) female(40-50) male(30-40) |
| 58 | (((0, 0), 1), ((0, 3), 1), ((1, 4), 1)) | 0.26% | 78 | female(0-10) female(30-40) male(40-50) |
| 59 | (((0, 0), 1), ((0, 3), 1), ((1, 2), 1)) | 0.26% | 77 | female(0-10) female(30-40) male(20-30) |
| 60 | (((0, 2), 1), ((1, 0), 1), ((1, 4), 1)) | 0.26% | 77 | female(20-30) male(0-10) male(40-50) |
| 61 | (((0, 1), 1), ((0, 3), 1), ((1, 4), 1)) | 0.25% | 74 | female(10-20) female(30-40) male(40-50) |
| 62 | (((0, 4), 1), ((1, 1), 1), ((1, 3), 1)) | 0.25% | 73 | female(40-50) male(10-20) male(30-40) |
| 63 | (((1, 3), 1), ((1, 5), 1)) | 0.25% | 73 | male(30-40) male(50-60) |
| 64 | (((1, 5), 1),) | 0.22% | 65 | male(50-60) |
| 65 | (((0, 0), 1), ((0, 3), 1), ((1, 0), 1), ((1, 3), 1)) | 0.21% | 63 | female(0-10) female(30-40) male(0-10) male(30-40) |
| 66 | (((0, 3), 1), ((1, 2), 1), ((1, 4), 1)) | 0.20% | 60 | female(30-40) male(20-30) male(40-50) |
| 67 | (((0, 4), 1), ((1, 2), 1), ((1, 6), 1)) | 0.19% | 57 | female(40-50) male(20-30) male(60-70) |
| 68 | (((0, 4), 1), ((1, 0), 1), ((1, 4), 1)) | 0.18% | 52 | female(40-50) male(0-10) male(40-50) |
| 69 | (((0, 1), 1), ((0, 5), 1)) | 0.17% | 51 | female(10-20) female(50-60) |
| 70 | (((0, 2), 1), ((0, 4), 1), ((1, 0), 1), ((1, 3), 1)) | 0.17% | 50 | female(20-30) female(40-50) male(0-10) male(30-40) |
| 71 | (((0, 5), 1),) | 0.17% | 50 | female(50-60) |
| 72 | (((0, 1), 1), ((0, 4), 1), ((1, 0), 1), ((1, 3), 1)) | 0.17% | 49 | female(10-20) female(40-50) male(0-10) male(30-40) |
| 73 | (((0, 0), 1), ((0, 4), 1)) | 0.16% | 49 | female(0-10) female(40-50) |
| 74 | (((0, 0), 1), ((0, 2), 1), ((1, 0), 1), ((1, 3), 1)) | 0.16% | 47 | female(0-10) female(20-30) male(0-10) male(30-40) |
| 75 | (((0, 2), 1), ((1, 2), 1), ((1, 4), 1)) | 0.15% | 45 | female(20-30) male(20-30) male(40-50) |
| 76 | (((0, 3), 1), ((0, 6), 1), ((1, 3), 1)) | 0.15% | 44 | female(30-40) female(60-70) male(30-40) |
| 77 | (((0, 4), 1), ((1, 2), 1), ((1, 4), 1)) | 0.15% | 44 | female(40-50) male(20-30) male(40-50) |
| 78 | (((0, 0), 1), ((0, 2), 1), ((1, 4), 1)) | 0.14% | 42 | female(0-10) female(20-30) male(40-50) |
| 79 | (((0, 1), 1), ((0, 3), 1), ((1, 0), 1), ((1, 3), 1)) | 0.14% | 41 | female(10-20) female(30-40) male(0-10) male(30-40) |
| 80 | (((0, 4), 1), ((1, 0), 1), ((1, 2), 1)) | 0.14% | 40 | female(40-50) male(0-10) male(20-30) |
| 81 | (((0, 3), 1), ((1, 3), 1), ((1, 6), 1)) | 0.13% | 39 | female(30-40) male(30-40) male(60-70) |
| 82 | (((0, 0), 1), ((0, 4), 1), ((1, 3), 1)) | 0.13% | 38 | female(0-10) female(40-50) male(30-40) |
| 83 | (((0, 0), 1), ((0, 3), 1), ((1, 1), 1), ((1, 4), 1)) | 0.13% | 37 | female(0-10) female(30-40) male(10-20) male(40-50) |
| 84 | (((0, 3), 1), ((0, 6), 1), ((1, 4), 1)) | 0.12% | 37 | female(30-40) female(60-70) male(40-50) |
| 85 | (((0, 2), 1), ((0, 5), 1), ((1, 0), 1), ((1, 3), 1)) | 0.12% | 36 | female(20-30) female(50-60) male(0-10) male(30-40) |
| 86 | (((0, 1), 1), ((0, 4), 1), ((1, 1), 1), ((1, 4), 1)) | 0.12% | 35 | female(10-20) female(40-50) male(10-20) male(40-50) |
| 87 | (((0, 1), 1), ((0, 3), 1), ((0, 4), 1)) | 0.12% | 35 | female(10-20) female(30-40) female(40-50) |
| 88 | (((0, 3), 1), ((1, 2), 1), ((1, 5), 1)) | 0.12% | 35 | female(30-40) male(20-30) male(50-60) |
| 89 | (((0, 2), 1), ((0, 4), 1), ((1, 1), 1), ((1, 4), 1)) | 0.12% | 35 | female(20-30) female(40-50) male(10-20) male(40-50) |
| 90 | (((0, 3), 1), ((0, 6), 1)) | 0.11% | 33 | female(30-40) female(60-70) |
| 91 | (((0, 2), 1), ((0, 4), 1), ((1, 3), 1)) | 0.11% | 33 | female(20-30) female(40-50) male(30-40) |
| 92 | (((0, 1), 1), ((0, 3), 1), ((1, 2), 1)) | 0.11% | 32 | female(10-20) female(30-40) male(20-30) |
| 93 | (((0, 4), 1), ((1, 2), 1), ((1, 5), 1)) | 0.11% | 32 | female(40-50) male(20-30) male(50-60) |
| 94 | (((0, 1), 1), ((0, 3), 1), ((1, 1), 1), ((1, 4), 1)) | 0.10% | 30 | female(10-20) female(30-40) male(10-20) male(40-50) |
| 95 | (((0, 1), 1), ((0, 4), 1), ((1, 2), 1)) | 0.10% | 29 | female(10-20) female(40-50) male(20-30) |
| 96 | (((0, 2), 1), ((1, 2), 1), ((1, 5), 1)) | 0.10% | 29 | female(20-30) male(20-30) male(50-60) |
| 97 | (((0, 1), 1), ((0, 4), 2)) | 0.10% | 29 | female(10-20) female(40-50) female(40-50) |
| 98 | (((0, 0), 1), ((0, 4), 1), ((1, 4), 1)) | 0.10% | 29 | female(0-10) female(40-50) male(40-50) |
| 99 | (((0, 2), 1), ((1, 0), 1), ((1, 3), 2)) | 0.10% | 28 | female(20-30) male(0-10) male(30-40) male(30-40) |
| 100 | (((0, 0), 1), ((0, 3), 2)) | 0.10% | 28 | female(0-10) female(30-40) female(30-40) |
