# Supplementary material for: Generating synthetic population for simulating the spatiotemporal dynamics of epidemics
Source: PLoS Comput Biol. 2024 Feb 12;20(2):e1011810. doi: 10.1371/journal.pcbi.1011810 (PMC10890746; doi:10.1371/journal.pcbi.1011810)
Supplement: S2 Table — The Peak Date, Peak Incidence Rate, and Attack Rate of the epidemic curve, including both the entire population and various age groups, are provided. Standard deviations (SD) and confidence intervals (CI) are also included. (DOCX) [file pcbi.1011810.s005.docx]

**Table S2. Numerical results of epidemic simulation based on different population synthesis methods**

| **Age** | **Synthesizer** | **Peak Date** | **Peak Incidence Rate(%)** | **Attack Rate(%)** | **STD of Peak Incidence Rate** | **STD of Attack Rate** | **CI of Peak Date** | **CI of Peak Incidence Rate** | **CI of Attack Rate** |
| --- | --- | --- | --- | --- | --- | --- | --- | --- | --- |
| Full-Population | MHO | 39(38,41) | 2.35(2.32,2.38) | 49.18(45.58,52.83) | 0.03 | 0.12 | 7 | 0.18 | 0.83 |
| Full-Population | DI | 39(38,41) | 2.35(2.27,2.44) | 49.19(44.31,54.18) | 0.10 | 0.99 | 7 | 0.57 | 5.54 |
| Full-Population | IPF | 40(39,42) | 2.35(2.31,2.38) | 49.21(45.33,53.12) | 0.03 | 0.12 | 6 | 0.21 | 0.87 |
| y0-10 | MHO | 37(36,39) | 2.84(2.75,2.94) | 56.54(51.40,62.08) | 0.08 | 0.40 | 9 | 0.52 | 2.26 |
| y0-10 | DI | 38(36,40) | 2.84(2.59,3.11) | 56.67(49.01,64.79) | 0.30 | 4.74 | 8 | 1.88 | 24.40 |
| y0-10 | IPF | 39(38,41) | 2.76(2.66,2.85) | 55.67(50.41,61.26) | 0.08 | 0.42 | 8 | 0.59 | 2.61 |
| y10-20 | MHO | 37(36,40) | 2.93(2.82,3.04) | 58.31(52.74,64.13) | 0.09 | 0.58 | 9 | 0.62 | 3.25 |
| y10-20 | DI | 38(37,41) | 2.90(2.62,3.23) | 57.86(49.79,67.39) | 0.37 | 6.32 | 10 | 2.12 | 31.69 |
| y10-20 | IPF | 39(38,41) | 2.83(2.72,2.94) | 57.44(51.73,63.43) | 0.09 | 0.58 | 9 | 0.59 | 3.48 |
| y20-30 | MHO | 39(38,41) | 2.25(2.20,2.30) | 47.51(43.79,51.33) | 0.04 | 0.23 | 8 | 0.27 | 1.62 |
| y20-30 | DI | 40(39,42) | 2.24(2.15,2.33) | 47.19(42.35,52.37) | 0.10 | 1.89 | 8 | 0.63 | 9.72 |
| y20-30 | IPF | 40(39,43) | 2.27(2.22,2.32) | 47.84(43.92,51.98) | 0.04 | 0.23 | 8 | 0.26 | 1.67 |
| y30-40 | MHO | 39(38,41) | 2.25(2.20,2.31) | 47.41(43.72,51.21) | 0.04 | 0.19 | 7 | 0.25 | 1.24 |
| y30-40 | DI | 40(38,42) | 2.24(2.15,2.35) | 47.24(42.34,52.47) | 0.12 | 1.93 | 9 | 0.83 | 11.46 |
| y30-40 | IPF | 40(39,42) | 2.26(2.22,2.31) | 47.63(43.69,51.67) | 0.04 | 0.19 | 9 | 0.28 | 1.24 |
| y40-50 | MHO | 39(38,41) | 2.29(2.23,2.34) | 47.89(44.06,51.92) | 0.05 | 0.27 | 8 | 0.29 | 1.48 |
| y40-50 | DI | 39(38,42) | 2.30(2.18,2.43) | 48.10(42.79,53.75) | 0.15 | 2.60 | 9 | 1.01 | 14.98 |
| y40-50 | IPF | 40(39,42) | 2.29(2.23,2.34) | 47.96(43.84,52.17) | 0.05 | 0.26 | 8 | 0.33 | 1.61 |
| y50-60 | MHO | 39(38,42) | 2.30(2.21,2.39) | 48.19(43.78,52.80) | 0.07 | 0.45 | 8 | 0.45 | 2.54 |
| y50-60 | DI | 40(38,42) | 2.29(2.11,2.48) | 47.90(41.70,54.61) | 0.21 | 3.86 | 12 | 1.25 | 19.92 |
| y50-60 | IPF | 40(39,43) | 2.30(2.21,2.38) | 48.21(43.69,52.96) | 0.07 | 0.45 | 9 | 0.50 | 2.79 |
| y60-70 | MHO | 37(36,40) | 2.59(2.44,2.76) | 52.03(45.81,58.64) | 0.12 | 0.82 | 10 | 0.91 | 5.03 |
| y60-70 | DI | 38(36,41) | 2.63(2.18,3.09) | 51.99(41.46,63.67) | 0.49 | 8.87 | 11 | 2.41 | 40.66 |
| y60-70 | IPF | 39(37,42) | 2.53(2.40,2.70) | 51.34(45.10,57.98) | 0.13 | 0.82 | 10 | 0.77 | 5.43 |
| y70-80 | MHO | 36(34,39) | 2.77(2.51,3.13) | 53.61(44.02,65.04) | 0.25 | 1.63 | 12 | 1.49 | 7.94 |
| y70-80 | DI | 37(35,41) | 2.82(2.05,3.69) | 53.95(36.55,74.10) | 0.98 | 16.63 | 15 | 5.59 | 85.80 |
| y70-80 | IPF | 38(36,41) | 2.72(2.41,3.02) | 52.54(42.90,63.93) | 0.25 | 1.61 | 13 | 1.79 | 8.66 |
| y80+ | MHO | 36(34,40) | 2.83(2.48,3.27) | 54.07(41.58,68.32) | 0.34 | 2.20 | 14 | 1.95 | 14.16 |
| y80+ | DI | 37(34,41) | 2.83(2.04,4.07) | 54.42(35.10,79.91) | 1.23 | 20.63 | 18 | 7.08 | 96.81 |
| y80+ | IPF | 38(35,41) | 2.74(2.39,3.19) | 52.79(40.88,67.08) | 0.33 | 2.15 | 14 | 2.12 | 13.72 |
